# Water ordering controls the dynamic equilibrium of micelle–fibre formation in self-assembly of peptide amphiphiles

**DOI:** 10.1038/ncomms12367

**Published:** 2016-08-24

**Authors:** Sanket A. Deshmukh, Lee A. Solomon, Ganesh Kamath, H. Christopher Fry, Subramanian K. R. S. Sankaranarayanan

**Affiliations:** 1 Center for Nanoscale Materials, Argonne National Laboratory, Lemont, Illinois 60439, USA; 2 Department of Chemistry, University of Missouri-Columbia, Columbia, Missouri 65211, USA

## Abstract

Understanding the role of water in governing the kinetics of the self-assembly processes of amphiphilic peptides remains elusive. Here, we use a multistage atomistic-coarse-grained approach, complemented by circular dichroism/infrared spectroscopy and dynamic light scattering experiments to highlight the dual nature of water in driving the self-assembly of peptide amphiphiles (PAs). We show computationally that water cage formation and breakage near the hydrophobic groups control the fusion dynamics and aggregation of PAs in the micellar stage. Simulations also suggest that enhanced structural ordering of vicinal water near the hydrophilic amino acids shifts the equilibrium towards the fibre phase and stimulates structure and order during the PA assembly into nanofibres. Experiments validate our simulation findings; the measured infrared O–H bond stretching frequency is reminiscent of an ice-like bond which suggests that the solvated water becomes increasingly ordered with time in the assembled peptide network, thus shedding light on the role of water in a self-assembly process.

## Abstract (web summary)

The role of water in the kinetics of the self-assembly process of amphiphilic peptides still remains unknown. Sankaranarayanan *et al*. have shown through computational study that water has a dual nature when dictating the mechanism and dynamics of self-assembly of peptide amphiphiles.

## Graphical abstract

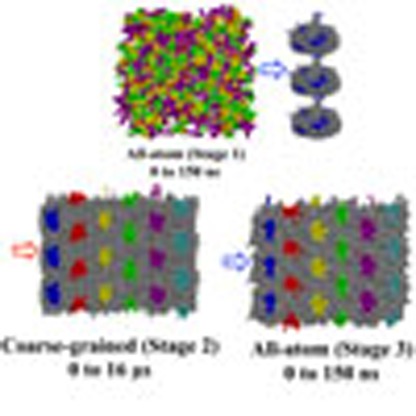

Peptide amphiphiles (PAs) which combine the structural features of amphiphilic surfactants with the functionality of bioactive peptides are emerging as promising candidates for avenues such as biomedical applications, biosensing, mineralization and as light-harvesting complexes^1^,^2^,^3^. Depending on the environmental conditions (pH, temperature, charge), PAs are known to self-assemble and form an entire gamut of structures—sheets, spheres, rods and disks^4^. One such common architecture resulting from the self-assembly of PAs is an ensemble of nanofibres that are typically microns in length and nanometre in diameter^3^. These fibrous assemblies of PAs have potential as scaffolds for light-harvesting fibres by ordering metalloporphyrins in a well-organized manner^5^. For example, in photosynthetic purple bacteria the distinguishing feature of the self-assembled structure of peptides and chlorophyll molecules is the well-organized multitude of chromophores^6^. This well-organized structure of chromophores facilitates the collection of sunlight, which can be utilized to catalyse the synthesis of useful fuel. To enable the design of a PA nanofibres with pre-defined well-ordered structure which can be used as a scaffold for light-harvesting fibres, it is important to understand the entire self-assembly process of these nanofibres.

Water is known to play an important role in the self-assembly of PAs, but their molecular details have often been thought to be secondary during the assembly process^7^,^8^,^9^. There have been a plethora of experimental as well as simulation studies based on atomistic, coarse-grained (CG), simple bead and packing models to study the structure and self-assembly of peptides^10^,^11^ and PA fibres^1^,^5^,^7^,^12^,^13^,^14^,^15^,^16^,^17^. In general, these studies were focused on understanding and exploiting the role of molecular interactions and secondary structure in PA self-assembly process. These studies primarily suggest that van der Waals and electrostatic interactions between the peptide molecules dictate the self-assembly process in PAs^13^. Despite recent spectroscopic data indicating that water has distinct, ordered behaviour near most biological interfaces, the exact role that water plays in the early stages of peptide assembly has remained elusive^18^. Given the recent success of atomistic and coarse-grained approaches in modelling self-assembly processes starting from a homogenous mixture of PAs solvated in water untill the formation of a single strand of fibre, it is logical to ask whether the chemical nature and local ordering of water play an important role during the assembly process? Furthermore, does water play any role in stabilization of the fibres before β-sheet formation between PAs, which have been considered to be vital in fibre formation^13^?

In the past, multistage-coupled atomistic and coarse-grained approach has been successfully utilized to study the protein folding, packing of DNA into viral capsids, and self-assembly of micelles^19^,^20^,^21^. For example, Brocos *et al*. employed MARTINI CG model to study the self-assembly of random mixtures of surfactants into micelles in explicit MARTINI water at the microsecond timescale^21^. They transformed the final CG configuration at the end of 5 μs back to all-atom model of micelles and performed all-atom molecular dynamics (MD) simulations for 250 ns. This approach allowed them to obtain the mean lifetime of the surfactant–solvent interactions as well as the lateral diffusion coefficients of the surfactant molecules within the micellar aggregates. Thus, the multiscale approach combines the advantages of all-atom and CG descriptions; while all-atom model allows one to investigate the structure at nanometre length and nanosecond timescales with angstrom-level and femtosecond resolutions, the CG model provides the capability for investigating the mesoscopic timescale (microseconds) and length scale (up to micron) dynamics that are critical to many biological and self-assembly processes.

Here, we embark upon a multistage-coupled atomistic and coarse-grained approach, complemented by experiments and advanced characterization to elucidate the solvation effects on the self-assembly process in c16-AHL_3_K_3_-CO_2_H PAs starting from randomly dispersed c16-AHL_3_K_3_-CO_2_H PAs into the formation of a bundle of assembled nanofibres. To successfully and accurately implement the multistage-coupled atomistic and coarse-grained approach, we consider factors such as: compatibility of all-atom and coarse-grained force-fields, mapping algorithm to transform an all-atom model to a CG model, back-mapping algorithm to transform the CG model to a fully atomistic model, and density of water in different regions of the structure in CG and all-atom models. By maintaining an atomistic resolution during the early stages of micelle and fibre formation to their subsequent aggregation into nanofibre bundles, we show that local interfacial ordering of water plays a key role in dictating the mechanism and dynamic equilibrium between the various phases during self-assembly of PAs. Our results brings to the forefront the dual functionality of water; breaking of interfacial ordering near the hydrophobic groups which causes the aggregation of PAs, and the ordered water in the vicinity of hydrophilic amino acids provide structure and order for the PAs thus enabling the formation of fibres. Our findings illustrate how the chemical nature and molecular details of aqueous interfaces control the early stages of PA assembly and provide quantitative insights into the unique role of water by drawing on the interfacial nature of hydration and aggregation kinetics associated with peptide assemblies.

## Results

### Mechanism of PA aggregation and micelle formation

**Figure 1:**
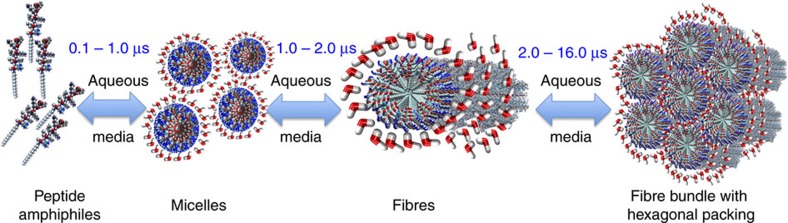
Schematic of the various stages involved in PA self-assembly. Text in blue shows the timescales taken for a completion of a particular stage in the present study.

**Figure 2:**
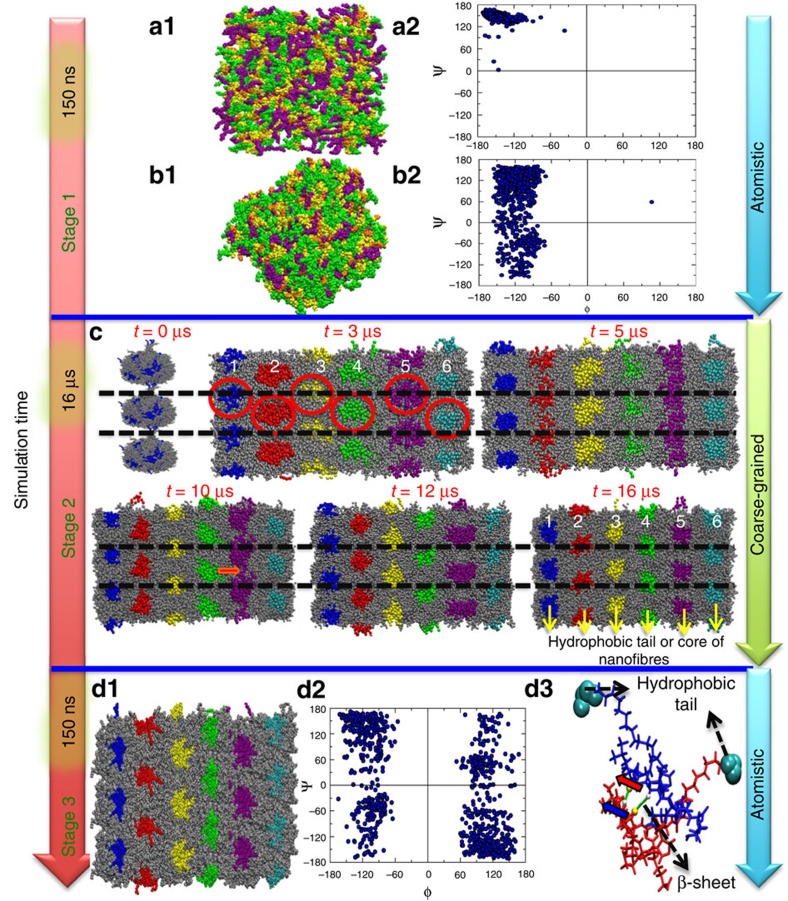
A multi-length-scale simulation showing the process self-assembly of PA nanofibres. (**a1**) and (**a2**) represent the all-atom models based on CHARMM force-field of PAs and Ramachandran plot for the starting configuration of randomly distributed PAs, respectively, and (**b1**) and (**b2**) represent equilibrated PAs configuration and Ramachandran plot at the end of 150 ns, respectively. Palmitoyl chains, Histidine, Leucine and Lysine are shown in purple, orange, yellow and green, respectively. Water molecules are removed for the purpose of clarity. (**b1**) and (**b2**) suggest no specific β-sheet formation at the end of ∼150 ns simulation run. (**c**) Snapshots showing the cross-section of PA fibres from 16 μs coarse-grained simulations. The early stage of self-aggregation and formation of bundles of nanofibres is shown in top panel. The red circles and white labels represent individual micelles and their respective indices. Hydrophobic tails or cores of six micelles and fibres are shown in blue, red, yellow, green, purple and cyan. The process of stabilization of hexagonal packing of PA fibres begins after 7.5 μs and fibre bundle remains intact. Red arrow shows the breaking of an individual fibre 5. Black dashed lines represent periodic boundaries in the *z*-direction. The hydrophilic groups are shown in grey. Fibre indices are shown in white at 3 and 16 μs. (**d1**) and (**d2**) show the final configuration and Ramachandran plot for the PAs based on the all-atom model at the end of ∼150 ns. (**d3**) shows the β-sheet formed between the two PAs at the end of the final ∼150 ns. Two PAs are shown in blue and green, respectively. The hydrogen and nitrogen atoms are shown in white and yellow, respectively, while the hydrogen bond between the β-sheet is shown in green. The hydrophobic tail is shown in cyan. The blue and red arrows show the β-sheet between the two peptides.

To study the various stages involved during self-assembly of PAs (Fig. 1), we monitor the self-assembly of PAs into bundles of nanofibres starting from an initial random homogeneous distribution (Fig. 2a1). We simulate the initial system with an all-atom model of PAs and explicit water molecules for 150 ns (stage 1; Fig. 2b1). Note, that the timescale of the self-assembly process of PA in experiments is on the order of milliseconds to minutes; therefore this study captures the atomistic picture of the earliest stages of self-assembly and micelle formation during PA self-assembly into nanofibres. In the initial phase of stage 1, the water molecules near the hydrophillic amino acids orient themselves to form hydrogen bonds with the polar atoms (*t*≥∼150 ps) (Supplementary Table 1). Further, in stage 1, we observe strong hydration around the hydrophobic region of the PA's palmitoyl tail (*t*≤∼150 ps) (Supplementary Fig. 1; Supplementary Table 2). In the vicinity of the hydrophobic alkyl chain, the water molecules re-organize themselves to form a hydrogen bond network with each other. This reorganization of water molecules near the hydrophobic tail leads to the formation of a water cage-like structure leading to an increase in the ordering of water around these hydrophobic groups (Supplementary Table 2). The breaking of the cage-like structure of water around the hydrophobic tail enables the hydrophobic collapse of the PA molecules and their subsequent aggregation driven primarily by van der Waals interactions between the hydrophobic groups of the PA. The formation and breaking of the cage of water molecules often called the hydrophobic effect has been observed in previous experimental and theoretical studies of various ions, polymers, bio-molecules and surfactants^22^,^23^,^24^,^25^,^26^,^27^,^28^. Further to quantify the enthalpic and entropic contributions for the free energy during the initial aggregation process of PAs, we calculated the potential of mean force (PMF)^29^,^30^,^31^. Our simulations suggest that in the initial aggregation stages, the entropic contributions are much favourable as compared with the enthalpic contributions in stabilizing the contact pair state of PAs (Supplementary Discussion; Supplementary Figs 2–4). This can be attributed to the stable cage-like structure of water near the hydrophobic groups of PAs.

During stage 1, water is expelled from the hydrophobic region of the PAs. This leads to a significant rearrangement and increased ordering of water molecules in the first solvation shell near hydrophilic groups (Supplementary Table 1). The hydrophobic and hydrophilic groups of PAs along with the vicinal water thus play different roles in the process of self-assembly; specifically, the hydrophobic groups cause the aggregation of PAs in water that eventually leads to the formation of micelles, while the hydrophilic groups that are in direct contact with water stimulate structure and order into the PAs to form fibres. Similar structural changes both in the structure of water and PAs have been reported by Yu and Schatz during the self-assembly process of PA nanofibres^32^.

Initial ∼150 ps and final ∼5 ns from a total of ∼150 ns trajectory was analysed to study the effect of structure of water near the hydrophobic groups of PA molecules on the self-assembly process. The reduction in average end-to-end distance of PAs from ∼25 ± 1 Å to ∼20 ± 1 Å owing to aggregation of the hydrophobic tails and removal of most of the water near hydrophobic tails marks the end of stage 1 in self-assembly (simulation timescale ∼150 ns). We map out the *ϕ*–*ψ* dihedral angles commensurate of the Ramachandran plot for the peptide backbone to track the β-sheet formation in the fibres during the self-assembly process (Fig. 2). In the beginning of stage 1 (*t*=0 to 10 ps), the distribution of the *ϕ*–*ψ* dihedral angles in the Ramachandran plot in Fig. 2a2 is extremely localized as all the molecules have identical conformations (Fig. 2a1). With increase in simulation time, we observe the spreading out of this population in the P_II_ region which corresponds to *ϕ*, *ψ*=−65°, +145° and extends over about 50° in both, *ϕ* and *ψ* dihedral phase space, respectively as shown in the Ramachandran plot (end of stage 1 (*t*=∼145 to ∼150 ns)) (Fig. 2b2 and the corresponding conformation is depicted in Fig. 2b1)^33^. In previous studies, the P_II_ region has been attributed to the enhancement of the entropy of the chain while exposing all backbone atoms that are capable of forming hydrogen bonds with water in a way that minimally disrupts the natural water organization^34^. Similarly, in the present study, we observe that the hydrophilic groups of PAs are exposed to water while water near the hydrophobic tails is expelled from the aggregate. Our simulations complemented by experiments probe the lag phase and report on the unique role of solvent in controlling the dynamic equilibrium between the micelle ←→ fibre phases by following the dynamical spatio-temporal evolution from an ensemble of micelles to a stable hexagonal network of the PA nanofibres.

### Self-assembly of PA nanofibres and their hexagonal architecture

We next coarse-grain the atomistic PA–water system obtained after the aggregation of peptides in all-atom model at the end of ∼150 ns (end of stage 1) to embark on a multi-length-scale simulation (stage 2 as depicted in Fig. 2c). A salient feature of this work relies upon a multi-length-scale simulation approach to study the self-assembly of PAs in fibres. The detailed mapping of the PA atoms with the CG beads is provided in Supplementary Table 3; Supplementary Fig. 5. The self-assembly process (0–1.5 μs) proceeds via aggregation of ∼50 PAs to form six different micelles, shown in red circles in Fig. 2c, thus presenting a three-dimensional architecture. This aggregate formation from individual PAs to micelle formation, as observed in our simulations, is in excellent agreement with the free energy considerations that suggests an aggregation number—*N*_agg_>40 PAs—which is typically required for the PAs to aggregate and form micelles^35^. All the six micelles are in close contact via van der Waals interactions. The two micelles present at each end, namely micelle number 1 and 6 (Fig. 2c), which are in direct contact with water first transform into cylindrical fibres along the *y* axis. Specifically, the Lysine, Leucine and Histidine amino acids of the micelles 1 and 6 are in close contact (within 15 Å) with the bulk water beads. Local ordering of water plays a crucial role in the initiation process of micelle coalescence and fibre formation (Fig. 4b3; Supplementary Discussion; Supplementary Methods provides details on the calculations of orientational order parameter).

**Figure 3:**
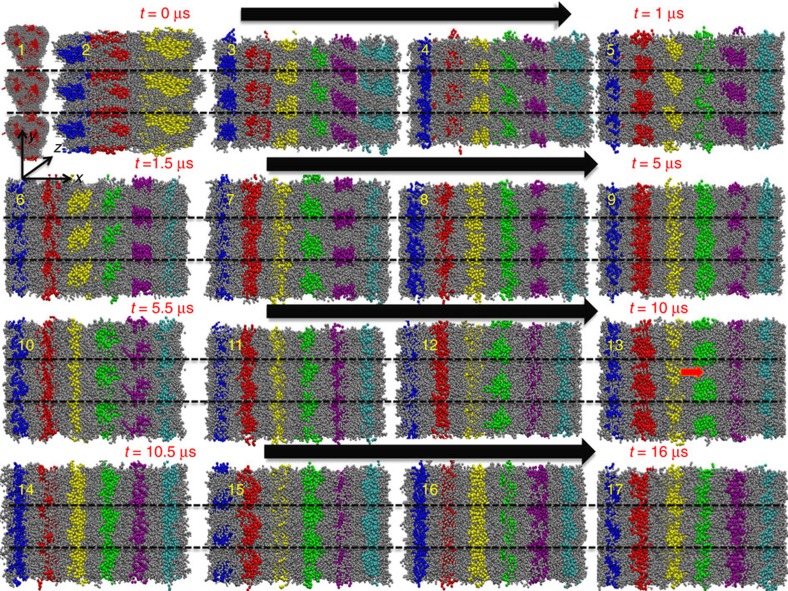
Dynamic process of fibre formation from a homogenous mixture. Snapshots show dynamical evolution from ∼0.1 to 16 μs during stage 2 of the fibre assembly process. The axial view (along the axis) of the fibre is depicted. Snapshot 1 shows the micelle formation (simulation time ∼0.1 μs). Snapshots 2 and 3 suggest fibre formation (simulation time >∼0.1 and <∼0.4 μs). Snapshot 4 shows the breaking of longer fibre into smaller micelles (simulation time >∼0.4 and <∼0.5 μs). Snapshots 5 to 7 show formation of fibres adjacent to bulk water beads (simulation time >∼0.5 and <∼0.8 μs). Snapshots 8 to 11 show formation of subsequent fibres (simulation time >∼0.8 and <∼3.0 μs). Snapshot 12 to 15 suggest breaking and formation of fibres (simulation time >∼3.0 and <∼12.0 μs). Snapshots 16 and 17 show stable fibres (simulation time >∼12.0 till ∼16.0 μs). Hydrophobic tails of six micelles and fibres are shown in blue, red, yellow, green, purple, and cyan. The hydrophilic groups are shown in grey. Water beads are not shown for clarity. Black line shows the periodic boundaries. Fibres 1, 2, 3, 4, 5, and 6 are shown in blue, red, yellow, green, purple, and cyan hydrophobic tails, respectively. Black arrow shows the increase in simulation time. Red arrow shows the breaking of fibres.

We observe that the fibres with indices 1 and 6 completely stabilize first, followed by micelles 2, 3 and 4 which transform into stable fibres within ∼2.5 μs, along the *y* axis. A hexagonal packing of these fibre bundles is evident at ∼3 μs (Fig. 2c). The process of fibre formation appears to be dynamic with a fibre breaking into micelles (two children micelles ∼25 PAs) and reuniting to form a fibre by the end of ∼12 μs (snapshot shown in Fig. 3). The hexagonal packing remains stable between ∼7.5 μs and ∼16 μs of CG MD simulations (Fig. 2c) and subsequently in stage 3 when the beads are atomistically resolved. While PA fibres with high concentration are known to bundle themselves and form close packed hexagonal network^36^, we demonstrate that water molecules between fibres in these close packed hexagon play a key role in stabilizing this fibre network. Note that the process of micelle formation followed by fibre formation has been reported in previous experimental and simulation studies^13^,^37^,^38^,^39^,^40^,^41^. Previously Tirrell *et al*. have investigated self-assembly of C16−W3K PA by using a suite of different experimental methods including cryogenic transmission electron microscopy (cryo-TEM), rheological measurement, circular dichroism, and atomic force microscopy (AFM) and infrared (IR) spectroscopy in the attenuated total reflection mode^39^,^40^. The cryo-TEM images of the C16−W3K solution taken immediately after making the solution showed existence of many discrete spherical micelle structures of around 10 nm diameter^39^. They also found spherical and shorter ‘worm-like' micelles which suggested that micellar transition occurs through collision of spherical micelles and/or shorter extended micelles; at least during the initial stages^40^. AFM images by Shimada *et al*. for multiple time incubation samples of (10, 20, 30, 60, 120 and 210 min) of C16-W3K PAs at 50 °C revealed fibril-like materials, that is, worm-like micelles, while many block-like materials were observed in 10 min incubation samples. The block-like material was presumed to be an aggregate of unstable spherical micelles that have no intermolecular hydrogen bonds during the process of drying. Further their AFM images of the samples before incubation exhibited block-like materials with no evidence of fibril-like structure; thus allowing them to infer that nucleation of elongated micelles and chain elongation of the micelles occur simultaneously during the assembly process. However, in our simulations, we report the dynamical evolution from an ensemble of micelles to a stable hexagonal network of the PA nanofibres.

### All-atom simulations of hexagonal architecture of nanofibres

The final configuration at the end of ∼16 μs CG MD simulation was transformed back into an all-atom model with modified TIP3P water molecules re-inserted (stage 3 in Fig. 2d1). The back-mapping process involves random placement of atoms near their corresponding coarse-grained bead followed by restraining the centre of mass of these atoms to the position of the coarse-grained bead^42^. Previously, such an approach has been used to atomistic back-mapping of simple peptides and transmembrane proteins from coarse-grained trajectories^43^. As mentioned earlier, our analysis of water beads during the CG MD simulation runs suggest the presence of strong ordering. Such an ordering is known to increase the density of water molecules in the first hydration shell present near biomolecules^44^. In addition, interaction of these water molecules with hydrophilic groups of PAs and steric hindrance created by the fibre network of PAs can significantly affect the diffusion of water molecules. To replicate this behaviour, we strategically re-inserted water molecules to maintain the higher density of ∼1.1 to ∼1.2 g cc^−1^ water between the two fibres. However, the overall water density was kept at 1 g cc^−1^. To ensure that the all-atom structure of both water and PA fibres is further relaxed, simulations of PA with CHARMM force-field and modified TIP3P water model were performed for ∼150 ns.

We discover that the self-assembled PA fibres after ∼150 ns retain the hexagonal packing with the hydrophobic tail in the fibre core (Fig. 2d1). From the Ramachandran plot and snapshots, we find that the dominant secondary structure observed in our simulations is the random coil-like structure and not the β-sheet (note: in the lower right quadrant of the Ramachandran plot, many of the amino acids changed from the L-conformation to the D-conformation upon converting from coarse-grained to atomistic simulations; Fig. 2d2). The Ramachandran plot is averaged over last 100 ps of total ∼150 ns simulation run of Stage 3. Similar coil-like structure has been previously reported by Lee *et al*^12^. Here, we also observe the formation of β-sheets parallel to the axis of fibre. We observe that the β-sheets are scattered around the inside of the nanofibre and do not show the continuous β-sheet structure along the fibre axis^12^,^45^. The presence of only a few examples of β-sheet interactions in the self-assembled structures could be partly due to the relatively smaller atomistic simulation timescales of ∼150 ns (Fig. 2d3).

### Mechanism of PA nanofibre formation

**Figure 4:**
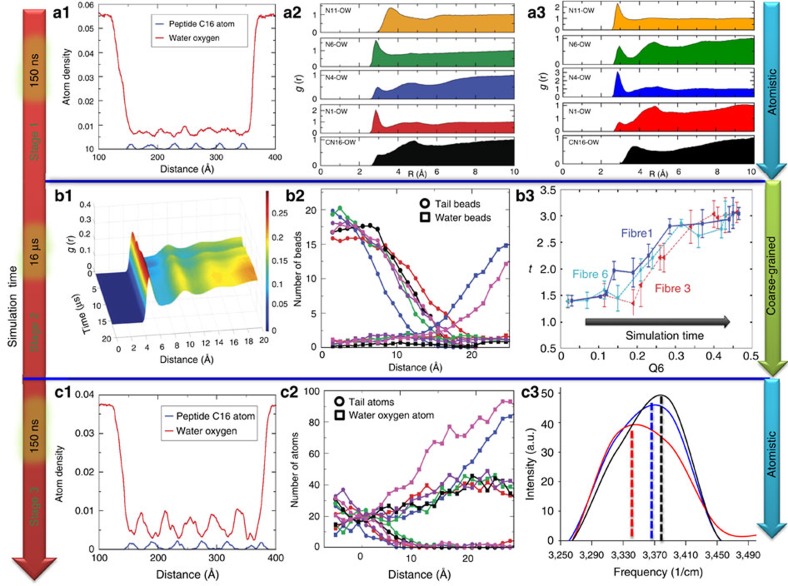
Role of local ordering and solvation effects in driving the self-assembly of PAs. Radial distribution function (RDF) (g(r)) of oxygen of water with CN16, N1, N4, N6 and N11 atoms of the hydrophobic tail, Alanine, Histidine, Leucine and Lysine amino-acid residues of PAs, respectively (Supplementary Fig. 1), (**a2**) after ∼500 ps and (**a3**) after ∼150 ns in stage 1. (**a1**) *X* axis density of water and C16 atom type of PAs at the end of ∼150 ns for CHARMM force-field in stage 1. *X*=0 refer to the minimum *X* axis value or in other words the minimum *X* axis coordinates of the box length. (**b1**) RDF (g(r)) of water beads with Lysine beads of the PAs of fibre 1 during 16 μs of simulation run. RDF suggests increase in ordering of water molecules with increase in simulation time. See Fig. 2 for fibre index. (**b2**) Number distribution of hydrophobic tail and water beads for coarse-grained model during the last 2 μs of the simulation in stage 2. (**b3**) The order parameters Q6 and *t* of water beads within 15 Å of fibres 1, 3 and 6 shown in blue, red and cyan, respectively. Ordering of water beads increases for all fibres as simulation progresses from 0.5 to 10 μs. After 10 μs water ordering is not affected as fibres 1, 3 and 6 are stabilized. Note, similar increase in ordering of water was observed for fibres 2, 4 and 5 with increase in simulation time. (**c1**) *X* axis density of water and C16 atom type of PAs at the end of ∼150 ns for CHARMM force-field in stage 3. Density profile of water and PAs also confirms the presence of small quantity water molecules into the PAs at the end of stage 3. (**c2**) Number distribution of hydrophobic tail and water molecules during last 10 ns of the simulation in stage 3. (**c3**) The stretching band of the vibrational spectra for hydrogen of water molecules at the end of stage 1 and stage 3 and for bulk water. The black, blue, and red lines represent bulk water, proximal water at the end of stage 1, and proximal water at the end of stage 3, respectively.

Figure 3 shows the axial view of the coarse-grain MD trajectories and reveals the dynamic equilibrium that exists between the micellar phase and fibre phase. For simulation time <0.8 μs as shown from snapshots 1–4 of CG MD simulation, the PA micelles are in dynamic equilibrium with the fibres (Supplementary Movie 1). This relative population of micelles and fibres are mainly controlled by the van der Waals interactions between PA and PA and between PA and water. The fibre formation in PAs coincides with the increased ordering of water near the hydrophilic groups of PAs, which suggest ordering of water facilitates the fibre formation (Fig. 4b3). At ∼0.8 μs, the micelles that are in direct contact with bulk water transform to form fibres first (snapshots 5 to 7). The formation of the fibre phase establishes equilibrium between fibres ←→ micelles (snapshots 8 to 15; Supplementary Movies 1 and 2). Between 1 and 12 μs, the fibres can be transformed back to micelles and gain stability only after water around the hydrophilic groups of the fibres is ordered (snapshot 16 and 17; Supplementary Movies 2 and 3). Subsequent to the fibre formation stage at times >12 μs and their arrangement into a hexagonal network, the bundle of fibres are stabilized via inter and intra van der Waals interactions between fibres (Supplementary Movies 3 and 4). During the process of fibre formation, we do not observe any evidence of β-sheet formation, which suggests existence of an alternate mechanism (possibly the ordering of water) that aids the initial stability during the fibre formation.

### Role of water in self-assembly of PA nanofibres

We closely examine the role of water using a series of molecular-level metrics on the overall trajectories and unravel the role of water in the PA assembly. In the first stage of the self-assembly, that is, stage 1 of atomistic simulation, we observe strong solvation around the hydrophobic region of the peptide amphiphile molecule's palmitoyl tail. Figure 4a1 shows the density profiles for oxygen atom of water and C16 atom types of PAs along the *X* axis. The density distribution of both the water and PAs at the end of stage 1 is very broad. The broadening of the peaks in the atomic density profile is a result of integration of the atomic density for all the various randomly oriented water and PA molecules and hence the peaks are not fully resolved. In addition, the density profile of water and PAs also confirms the presence of a small quantity of water molecules inside the PAs at the end of ∼150 ns. This could be the water that is mainly present near the hydrophilic amino-acid residues (Alanine, Histidine, Leucine and Lysine) of PA.

Radial distribution function (RDF) between the oxygen of water and the hydrophobic alkyl tail of PA and between the oxygen of water and hydrophilic amino-acid residues of PA shows two well-defined peaks at ∼2.85 and ∼5.0 Å, respectively, indicative of strong hydrogen bonding interactions between water and hydrophilic groups of amino acids (Fig. 4a2). The water molecules near the O1 of Alanine, N4 of Histidine, O5 of Leucine, O9 and N11 of Lysine orient themselves to form a hydrogen bond with these polar atoms (Supplementary Fig. 1; Supplementary Table 1). As seen in Fig. 4a2 and Supplementary Fig. 1; the structure of water is substantially different near the tail atoms when compared with those in the vicinity of the amino acids. For the case of the alkyl chain, the water molecules re-organize themselves to form a hydrogen bond network with each other. This reorganization of water molecules near the hydrophobic tail would lead to the formation of a water cage-like structure and increased ordering of water around these hydrophobic groups (Supplementary Fig. 1; Supplementary Table 1).

The breaking of the cage-like structure of water around the hydrophobic tail appears to be a rate-limiting step before the hydrophobic collapse of the PA molecule that yielded micelles. Cage breakage is suggested by the decrease in the first neighbouring peak and the second broad peak disappears which indicates reduction of water ordering in the second hydration shell near the PA (Fig. 4a3; Supplementary Table 1). This is consistent with prior observations of biomolecule solvation, where water cages form around the hydrophobic regions of peptides due to the lack of available polar regions^46^. The water cage stability can be altered by changing the temperature and/or adding salt ions (varying the pH of the system)^47^. The breaking of the water cage around the hydrophobic tails exposes them to one another, which leads to hydrophobic collapse of the PAs and water being expelled from the hydrophobic region of the PAs, resulting in significant rearrangement of water molecules in the first solvation shell.

After hydrophobic collapse, stage 2 highlights the PA micelles fusing together to ultimately form a bundled network of fibres. The RDF of water beads with Lysine amino-acid beads of fibre 1 is shown in Fig. 4b1. The 1st and 2nd peaks can be observed at ∼5.05 and ∼9.95 Å, respectively. The peak height for both 1st and 2nd peaks increases with an increase in simulation time. This suggests an increase in ordering of water molecules near the hydrophilic groups of PAs, enabling the stabilization of fibres during the process of bundle formation. This is corroborated by the order parameter calculations of water beads over the 16 μs of CG MD simulations where we find that orientational order parameter (Q_6_) of the water beads increasing with increase in simulation time (Fig. 4b3). The water beads near fibre 1 and fibre 6 order before the water near interior fibres (fibre 3 for example in Fig. 4b3) of the hexagonal network (see Fig. 2 for definition of fibre 1 to 6). This ordering of water beads could stabilize fibres 1 and 6 before the rest of the fibres in the hexagonal network.

The distribution of water beads over the last 3 μs of total 16 μs CG MD simulations is shown in Fig. 4b2. The bead distribution was calculated from the core of a fibre. A radius of cylindrical PA fibre is shown in Supplementary Fig. 9. In the case of end fibres (fibres 1 and 6), (see Fig. 2 for definition of fibre 1 to 6), the degree of solvation increases as we go away from the hydrophobic tail. In the case of intermediate fibres (2, 3, 4 and 5), we do observe the presence of a small number of water beads. This could be due to the close packing of these fibres that restricts both the number and mobility of water beads within the closely packed hexagonal structure. Increase in ordering of these water molecules near the PA fibres might stabilize the fibres and bundle of these fibres. In all the six fibres, the tails aggregate inside the fibre due to hydrophobic collapse/interactions and the concentration of tail group atoms is significantly higher within 10 Å from the core of the fibres.

In stage 3, after the fibres have assembled into bundles, the atom density contour at the end of ∼150 ns exhibits characteristic contours ∼50 Å apart (Fig. 4c1). This is representative of self-assembled PA fibres with a ∼50 Å diameter which is consistent with our previous experimental measured diameter of ∼69 Å for fully inter-digitated alkyl tails^5^. Each fibre of the self-assembled hexagonally packed structure consists of ∼50 PAs with ∼11 PA per nm along the fibre axis consistent with the single fibre self-assembly results of Lee *et. al.* (∼14 PA per nm along the fibre axis and the diameter of fibre is ∼80 Å)^13^.

The close packing of the fibres restricts both the number and mobility of water beads within the closely packed hexagonal structure (Fig. 4c2). The water density contours show well-defined and distinct contours, which suggest a strong ordering (Fig. 4c1). Indeed, the decrease in the tetrahedral order parameter near the hydrophilic groups of fibres suggests increase in ordering of water molecules near fibres (Supplementary Table 4). In addition, we find that the translational order increases for water molecules near the hydrophilic groups of fibres as compared with water molecules away from the fibres (Supplementary Table 4; Supplementary Fig. 8). This presence of ordered water in the self-assembled PA nanofibres is comported by experimental studies of Tovar *et al*.^14^ where they found the interior of the PAs to be well-solvated. During the micelle ↔ fibre transformation, the locally ordered water ensures that van der Waals interactions between PAs and water play a major role in formation of stable fibres. In another study, Yu and Schatz^32^ combined targeted molecular dynamics simulations, umbrella sampling and the weighted histogram analysis method, to calculate the PMF for the transition between the bound and free states of 90 PAs in aqueous solution. The bound state represented a cylindrical micelle–fibre. In particular they have studied the process of the disassembly of PA fibres from bound state to free state as a function of change in the *R*_g_ of the PA nanofibres. They observed that during the disassembly process, in addition to structural changes in the PA nanofibres, the water molecules also undergo a large redistribution to adapt to the PA structural changes in the bound-to-free state transition process.

The characteristic structural features observed at the atomic scale for the ordered proximal water molecules in the PA nanofibres are strongly correlated to their vibrational densities of states (Supplementary Method; Supplementary Discussion). The vibrational spectra of water molecules were calculated by Fourier transforming of the atomic velocity autocorrelation function obtained from the MD simulation trajectories in stage 3. The stretching band of vibrational spectra for hydrogen of water molecules in bulk and for proximal water molecules (within 5 Å of PA molecules) at the end of ∼150 ns simulation run of stage 1 and stage 3 is shown in Fig. 4c3 (Supplementary Discussion). Experimentally, the O–H stretching band of the proximal water is known to show a red shift because of the presence of the strong hydrogen bonds^48^,^49^. We find that the O–H stretching band for proximal water at the end of stage 3 shows a prominent red shift at ∼3,340 cm^−1^as compared with ∼3,370 and ∼3,390 cm^−1^ for proximal water at the end stage 1 and bulk water, respectively. This red shift in O–H stretching band can be attributed to the increase in the strength of hydrogen bonds due to increased ordering of proximal water at the end of stage 3 (Supplementary Table 4). The presence of ordered water can form the hydrogen bond bridges between water and water, which could lead to formation of very strong and stable hydrogen bonds between PA and water (Supplementary Fig. 8). Similarly, the bending and libration band of hydrogen (Supplementary Fig. 10) and the vibrational spectra of oxygen of water (Supplementary Fig. 11) strongly support the notion of strong hydrogen bond network of proximal water molecules near PA molecules at the end of stage 3 (Supplementary Discussion).

Residence time calculations suggest that the water molecules near the hydrophilic regions of PA reside much longer in comparison to the water molecules near the hydrophobic region indicative of more stable water structures near hydrophilic regions (Supplementary Discussion; Supplementary Fig. 6).^28^ This can be attributed to the presence of water molecules in the PA network (Supplementary Fig. 8) that form stronger hydrogen bonds with the amino acids of PA, assisting water molecules to form a stable network near the hydrophilic region (Supplementary Discussion; Supplementary Fig. 7).^49^ Note that in spite of fibre formation, we did not observe a significant amount of β-sheet content in these networks when the coarse-grain configuration of stage 2 were converted back to fully atomistic in stage 3. Typically, previous computational modelling and simulation of PAs focuses on the hydrophobic collapse and monitor β-sheet formation, although it was suspected that β-sheets, while they do strengthen fibre assemblies, are not required in the formation of fibres. Here, we find that the water molecules assist the PAs to form and stabilize the long range ordering of β-sheets.

### Experimental validation of solvation effects

**Figure 5:**
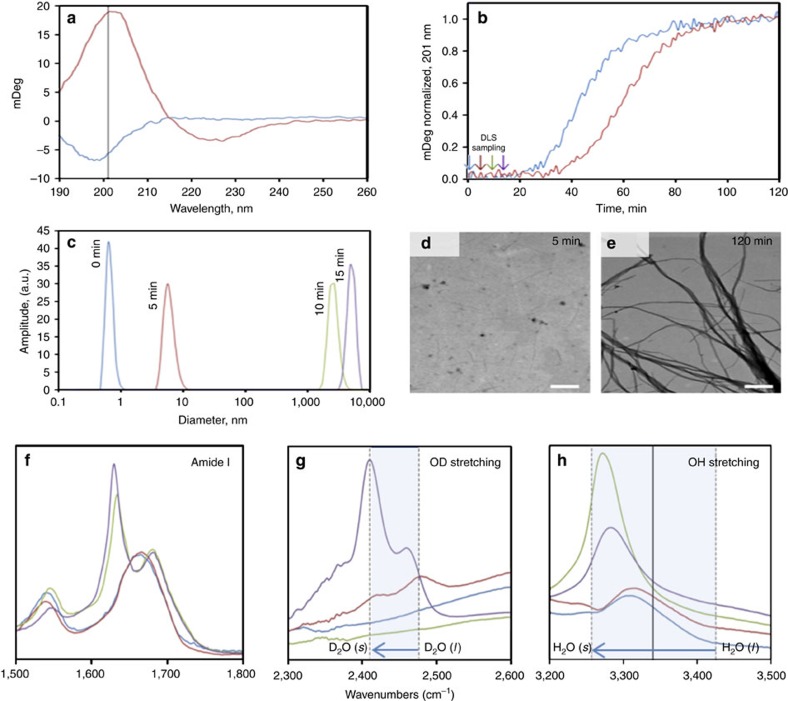
Experimental characterization of structure of water during self-assembly of PA nanofibres. Aggregation kinetic studies to probe the role of water during the various stages from an initial micelle to early stages of formation of PA fibres are shown in **a**–**e**. (**a**,**b**) Aggregation kinetics monitored by β-sheet formation. (**a**) Circular dichroism spectra of c16-AHL_3_K_3_-CO_2_H measured in water (blue) and 100 mM ammonium hydroxide after 120 min (red). Vertical solid line at 201 nm marks where the kinetics were monitored. (**b**) Aggregation kinetics of β-sheet formation monitored at 201 nm in 100 mM ammonium hydroxide (blue) and 70% D_2_O in 100 mM ammonium hydroxide (red). Arrows mark times wear DLS samples were monitored in the absence of β-sheets. (**c**) Aggregation size with respect to time. Dynamic light scattering measurements at times: 0 min, blue; 5 min, red; 10 min, green; 15 min, brown. (**d**,**e**) Fibre morphology variation with respect to time. Electron micrographs of c16-AHL_3_K_3_-CO_2_H fibres at: (**d**) 5 min and (**e**) 120 min, scale bar, 300 nm. (**f**–**h**) Experimental infrared spectra highlighting the amide I region (**f**), the O–D stretching region (**g**) and the O–H stretching region (**h**). c16-AHL_3_K_3_-CO_2_H in H_2_O (blue line), 70% D_2_O/30% H_2_O (red line), 5 mM NaOH (green line), and 5 mM NaOH in 70% D_2_O/30% H_2_O (purple line). Frame (**g**) highlights the range of D_2_O (l) from ∼2,476 cm^−1^ to D_2_O (s) ∼2,410 cm^−1^. Frame (**h**) highlights the range of H_2_O (l) from ∼3,425 cm^−1^ to H_2_O (s) ∼3,257 cm^−1^. Frame (**h**) also shows the value for computational vibrational spectra for proximal water at ∼3,340 cm^−1^ (vertical black line).

Experimental determination of PA dynamics is of great interest in both early^50^ and late stage assembly^51^. To experimentally validate the solvation effects found in our computations and pairing it with the lack of observed β-sheets, we monitored the aggregation kinetics of the PA assembly in water and deuterium oxide (Fig. 5a–e). The postulation is that the deuterium oxide measurements will form a stronger solvation cage around the PA molecule slowing down the lag phase of the aggregation kinetics. Assembly is triggered by neutralizing the positively charged lysine head groups with ammonium hydroxide (100 mM final concentration, pH ∼11) thus eliminating electrostatic repulsion while facilitating intermolecular interactions. Fig. 5a,b show the results of the aggregation kinetics as monitored by circular dichroism spectroscopy. Over a 120 min period, we observe typical aggregation kinetics when monitoring β-sheet formation: a lag phase that is followed by a sharp increase in β-sheet content until β-sheet formation is complete. In H_2_O (100 mM ammonium hydroxide), the lag phase persists for about 20 min. When the solvent system is changed by strengthening the solvent cage around the PA molecule with 70% D_2_O (100 mM ammonium hydroxide), we observe an extension of the lag phase to 35 min. However, with dynamic light scattering, we observe the increase in aggregation size from sub-nanometres in dimension (that is, individual PA molecules) to microns in dimension during this measured lag phase, Fig. 5c. This would suggest that something larger than micelles is forming during this lag phase time period observed in the circular dichroism spectroscopy.

Using the TEM, we were able to observe that samples analysed at 5 min during the reaction time already formed fibres in spite of the fact that β-sheets were not observed (Fig. 5d). This supports the notion that β-sheets are not required for successful fibre formation when engineering these peptide materials. However, after the completion of β-sheet assembly for this particular PA, we observed enhanced bundling and a possible increase in fibre diameter consistent with a more rigid PA assembly seen in Fig. 5e. We conclude that the earliest stage, the lag phase (0–20 min), of the aggregation kinetics is experimentally relevant to our simulation results due to the fact that fibrous aggregates are observed as early as 5 min where β-sheets have yet to be formed. Other efforts towards studying early stage assembly highlight the lack of β-sheet structure in spite of the presence of supramolecular structure^50^. Therefore, both the experimental results and simulations agree that fibre pre-assembly occurs before the formation of extended β-sheet rich, fibre networks and that this early pre-assembly state is heavily reliant on the breaking of a water solvation cage followed by hydrophobic collapse.

## Discussion

Finally, we directly characterize the progression of water becoming increasingly ordered with respect to the hierarchical assembly by analysing the infrared vibrational spectra of the peptide in the monomer ←→ micelle equilibrium and the fully assembled β-sheet containing fibre (Fig. 5f–h). Note, to ensure the observed frequencies were a result of confined water and not simply peptide vibrations we prepared samples in both H_2_O and 90% D_2_O/10% H_2_O and compared the sample when it was dried under ambient conditions versus dried at 100 °C under reduced pressure (Supplementary Fig. 12; Supplementary Discussion). In addition, our thermogravimetric analysis suggested 7–10% loss of mass in the assembled fibres case and negligible loss in the unassembled-micelles case when heating to 200 °C (Supplementary Fig. 13; Supplementary Discussion). Experimentally and computationally the O–H stretching band for water and ice has been observed in the range of ∼3,425 to ∼3,206 cm^−1^, respectively^52^,^53^. Amorphous solid ice yields an O–H stretching band at ∼3,257 cm^−1^ (ref. 52). In the case of D_2_O, a similar red shift with ordering from liquid D_2_O to solid D_2_O with stretching bands at, *ν*=∼2,476 and ∼2,410 cm^−1^, respectively, has been reported^53^,^54^. We find that in the sample that represents the monomer ←→ micelle equilibrium, no specific secondary structure was observed as indicated by the broad amide I vibration at ∼1,665 cm^−1^ (Fig. 5f) in conjunction with the lack of a defined circular dichroism spectra, Fig. 5a (blue spectrum)^55^. In spite of this lack of defined secondary structure, a micelle structure has been observed. As seen in our computational study, micelles should yield a trapped network of water proximal to the peptide surface (Fig. 4). In addition, the computational vibrational spectra at the end of stage 1 shows a red shift in the stretching band of hydrogen of proximal water at ∼3,370 cm^−1^ as compared with ∼3,390 cm^−1^ of bulk water (Fig. 4c3). Similarly, in our experiments, we find a weak signal at *ν*=∼3,310 cm^−1^ (∼2,420 and ∼2,475 cm^−1^ for D_2_O) significantly red shifted from the experimental bulk water value of *ν*=∼3,425 cm^−1^ (∼2,476 cm^−1^ for D_2_O) (Fig. 5h). After heated vacuum drying, we observe a slight loss in absorption at ∼3,310 cm^−1^ in the case of H_2_O but in the case of D_2_O we observe a dramatic loss in ∼2,420 and ∼2,475 cm^−1^ with a rise at ∼3,300 cm^−1^ while the amide I vibrations remain largely unchanged. This observation confirms that the assignment of the vibrational mode is associated with trapped water.

Furthermore, analysis of the β-sheet containing fibres yield amide I bands at ∼1,631 and ∼1,681 cm^−1^ typical of a β-sheet assembly (Fig. 5f).^55^ As a result of the formation of the β-sheet rich fibres, we find that the ∼3,310 cm^−1^ band (∼2,480 and 2,420 cm^−1^ for D_2_O) red shifts to a lower frequency, ∼3,270 cm^−1^, (∼2,410 cm^−1^ for D_2_O) with greater intensity (Fig. 5g,h). After heated vacuum drying, we observe a loss of the signal at ∼3,270 cm^−1^ (∼2,410 cm^−1^ for D_2_O) suggesting a loss of trapped H_2_O (or D_2_O) while the β-sheet signals remain (Supplementary Fig. 12). These lower frequencies, obtained at room temperature, are close to that measured for ice suggesting perhaps that the trapped water becomes increasingly solid-like with increased structural order in the assembled peptide network. Note, in our computations we find a similar red shift from ∼3,370 cm^−1^ at the end of stage 1 to ∼3,340 cm^−1^ at the end of stage 3 that is attributed to trapped water. The difference in the computational and experimental values can be attributed to the longer experimental times. Thus, our experiments and computational study greatly complement one another in the description of water's important role in the ordering and hierarchical assembly of peptide amphiphiles from monomers to micelles to fibres.

In conclusion, we have performed multistage coupled atomistic-CG MD simulations to study the self-assembly of a homogeneous mixture of PA molecules into a hexagonal network of nanofibre bundles. Our simulations supported by experiments elucidate the crucial role of water molecules in facilitating the self-assembly in PAs. Breakage of ordered cages around the PAs leads to hydrophobic collapse, which leads to micelle formation. The observed role of the water cage was supported by the extended lag phase when D_2_O was employed as the solvent in our kinetic measurements suggesting monomer ↔ micelle ↔ fibre equilibrium. Water ordering controls the fusion dynamics of the micelles to form PA fibres. By analysing the overall trajectories of both CG and all-atom simulations and confirming experimentally (*vide infra*), we show that β-sheet content is not crucial to the initial fibre aggregation assembly mechanism. While such a secondary structure plays a role in the propagation and stability of larger fibrous structures that are capable of binding and encapsulating a photoactive chromophore, we find that the local ordering and solvation effects of water drive the initial fibre aggregation assembly mechanism and also provide stability to the network of assembled fibres^8^. We also find a great level of reassurance in our computational studies that extended β-sheet networks are not observed but packed fibrous networks are consistent with our microscopy studies measured during the lag phase before β-sheet formation. Furthermore, the coupling of the theoretical, proximal O-H vibrational frequency analysis with the vibrational spectroscopy measurements confirm and highlight the significance of water ordering in the hierarchical self-assembly of peptide amphiphiles. Our results provide extensive insights into early fibre formation and the role of solvent in the early lag phase component of the kinetics.

## Methods

### Computational methods

In the present study, we embark on studying the self-assembly of a model peptide nanofibre that is, the c16-AHL_3_K_3_-CO_2_H PA sequence which has emerged to be an ideal candidate for binding of metalloporphyrin light-harvesting molecules. This PA consists of a polar head, with three lysine residues, and a nonpolar, aliphatic tail, palmitic acid. It is known that the binding of metalloporphyrin occurs through a single histidine amino acid. Note that the peptide sequence plays an important role on the local packing of the molecule that eventually translates into high levels of organization in the supramolecular assembly. For this model PA system, therefore, it is important to understand the nature of binding pocket formed during the self-assembly stage and the resulting photophysical effects in the supramolecular arrangement^5^. The process of self-assembly of neutralized PAs was studied in three stages. In stage 1, a fully atomistic model of 300 units of c16-AHL_3_K_3_-CO_2_H PA molecules was generated and randomly distributed along with the modified TIP3P water model in the simulation cell^29^. The CHARMM community has effectively used modified TIP3P water model to determine the structural and dynamic properties of proteins, nucleic acid and carbohydrates. The determination of the partial charges on the various polar sites on the model compounds which are the building blocks for such macromolecules are based on the reproduction of quantum mechanical energy calculations and using modified TIP3P water model as a probe. Lysine molecules were neutralized by using –NH_2_ groups. The simulation steps involved a fully atomistic simulation (stage 1) followed by coarse-grained simulations (stage 2) and finally returning back to atomistic simulations (stage 3). For the fully atomistic molecular dynamics simulations, the CHARMM force-field with modified TIP3P water model was used to govern the interactions of peptide and water, while the MARTINI force-field was used for coarse-grained simulations^56^,^57^. The PA molecules were mapped into MARTINI CG model using visual molecular dynamics VMD. The solvate command of VMD was used to insert MARTINI water beads. VMD's Coarse-Grain Builder tool was used to back-map from the coarse-grained model to fully atomistic model^58^,^59^.

All-atom simulations were carried out in the isobaric-isothermal (NPT) ensemble for 150 ns with a 1 fs timestep^56^. Nanoscale Molecular Dynamics programme (NAMD) simulation package was used to perform the MD simulations^60^. Temperature and pressure were maintained at 300 K and 1 atm using the Langevin thermostat and barostat, respectively^61^,^62^. The relaxation time for thermostat and barostat were set to 50 and 100 fs, respectively. The particle-mesh Ewald method was used with a 1 Å grid width to calculate the electrostatics interactions^63^. A switching function was applied for all Lennard-Jones interactions at 12.5 Å for a 14.0 Å cut-off^56^. A smooth switching function was used to truncate the van der Waals potential energy smoothly at the cutoff distance. A switch distance of 10 Å was used for activating the splitting function for electrostatic and van der Waals calculations. A pair list distance of 14 Å was used to include the pairs of atoms for which electrostatics and van der Waals interactions were calculated. Simulation trajectories consisting of atomic coordinates and velocities were stored every 1 ps.

CG MD simulations were performed with the NAMD simulation package with a timestep of 30 fs in the NPT ensemble and the total simulation time was 16 μs. The pressure and temperature are controlled by employing the Langevin barostat and thermostat at 1 bar and 340 K, respectively. The neighbour list cutoff of *r*_cut_=2.0 nm was used and the list was updated every 10 steps. Simulation trajectories including coordinates and velocities of atoms were saved every 30 ps for the trajectory analysis. Additional force-field and simulation details can be found in Supplementary Methods.

### Experimental methods

The synthesis, purification and characterization of c16-AHL_3_K_3_-CO_2_H has been described in our previous work. Kinetic analysis of fibre assembly was performed on samples that were prepared by diluting a stock solution of c16-AHL_3_K_3_-CO_2_H in water (1 wt%, 8.4 mM) to 100 μM (3.6 μl of the peptide stock solution was added to 270 μl of either H_2_O (MilliQ Advantage A10, 18.2 MΩ) or D_2_O (Sigma-Aldrich, sealed ampule)). At this point, the solution was transferred to a quartz cuvette (1 mm path length) and was analysed for β-sheet content by using circular dichroism spectroscopy (JASCO J-815 circular dichroism Spectrometer) and/or aggregation by using dynamic light scattering (Malvern Instruments, Zetasizer Nano-ZS). The assembly was then triggered by adding 30 μl of an ammonium hydroxide solution (1 M) to trigger assembly. The final concentration of ammonium hydroxide was 100 mM. Circular dichroism was employed to monitor the formation of the positive n−π* transition (*λ*=201 nm) typical of β-sheets over a time period of 120 min while dynamic light scattering measurements were performed every 5 min until aggregation was complete.

The assembly was also monitored at two time points, 5 and 120 min. Samples (10 ml) were removed directly from the cuvette and dropcast onto a TEM grid (400 copper mesh with an ultrathin carbon layer, Ted Pella). After 30 s, the excess sample was wicked away from the TEM grid with filter paper. In the case of the 5 min sample, two grids were prepared where one was left unstained and the other was stained by dropcasting 10 ml of a filtered 1 wt% solution of phosphotungstic acid in water directly onto the grid and then wicked away with filter people after a 30 s exposure time. The samples were analysed using a JEOL 7500 FE-SEM (Accelerating Voltage, 30 keV; Emission Current, 10 μA) using the transmission electron detector.

Infrared spectroscopic measurements were made on a Thermo Scientific Nicolet 6700 FT-IR spectrophotometer. Samples were prepared by diluting a stock solution (1 wt%, 8.4 mM) of c16-AHL_3_K_3_-CO_2_H in water to 0.84 mM, 10 μl of the peptide stock solution was added to 90 μl of either H_2_O or D_2_O. β-Sheet assembly was triggered by addition of 1 μl of a 1 M NH_4_OH stock solution to yield a 10 mM final NH_4_OH concentration. Ultimately, four samples were analysed: c16-AHL_3_K_3_-CO_2_H in (1) H_2_O, (2)90% D_2_O/10% H_2_O, (3) 10 mM NH_4_OH and (4) 10 mM ND_4_OD in 90% D_2_O/10% H_2_O. Samples (10 μl) were dropcast onto a CaF_2_ plate (32 mm round cell window, Sigma-Aldrich) and were air-dried yielding thin transparent films. After FT-IR analysis of samples dried under ambient conditions, the CaF_2_ plates were transferred to a vacuum oven where they were heated at 100 °C under reduced pressure for 16 h.

### Data availability

The data that support the findings of this study are available from the corresponding authors upon request.

## Additional information

**How to cite this article:** Deshmukh, S. A. *et al*. Water ordering controls the dynamic equilibrium of micelle–fibre formation in self-assembly of peptide amphiphiles. *Nat. Commun.* 7:12367 doi: 10.1038/ncomms12367 (2016).

## Supplementary Material

Supplementary Information Supplementary Figures 1-13, Supplementary Tables 1-4, Supplementary Discussion, Supplementary Methods, and Supplementary References

Supplementary Movie 1 Micelle Formation. The movie demonstrates the micelle formation from homogenous mixture of PAs in water during initial 1μs of CG MD simulations. Hydrophobic tails of six micelles and fibers are shown in blue, red, yellow, green, purple, and cyan. Note, water is not shown for the purpose of clarity.

Supplementary Movie 2 Micelle to Fibers. The movie demonstrates the micelle to fiber transformation of PAs in water during 1 to 5 μs of CG MD simulations. Hydrophobic tails of six micelles and fibers are shown in blue, red, yellow, green, purple, and cyan. Note, water is not shown for the purpose of clarity.

Supplementary Movie 3 Fibers Stabilization. The movie demonstrates the micelle to fiber transformation of PAs in water during 5 to 10 μs of CG MD simulations. Hydrophobic tails of six micelles and fibers are shown in blue, red, yellow, green, purple, and cyan. Note, water is not shown for the purpose of clarity.

Supplementary Movie 4 Stabilized Fibers. The movie demonstrates the micelle to fiber transformation of PAs in water during 10 to 16 μs of CG MD simulations. Hydrophobic tails of six micelles and fibers are shown in blue, red, yellow, green, purple, and cyan. Note, water is not shown for the purpose of clarity.
