# Supplementary material for: Water ordering controls the dynamic equilibrium of micelle–fibre formation in self-assembly of peptide amphiphiles
Source: Nat Commun. 2016 Aug 24;7:12367. doi: 10.1038/ncomms12367 (PMC4999504; doi:10.1038/ncomms12367)
Supplement: Supplementary Information — Supplementary Figures 1-13, Supplementary Tables 1-4, Supplementary Discussion, Supplementary Methods, and Supplementary References [file ncomms12367-s1.pdf]

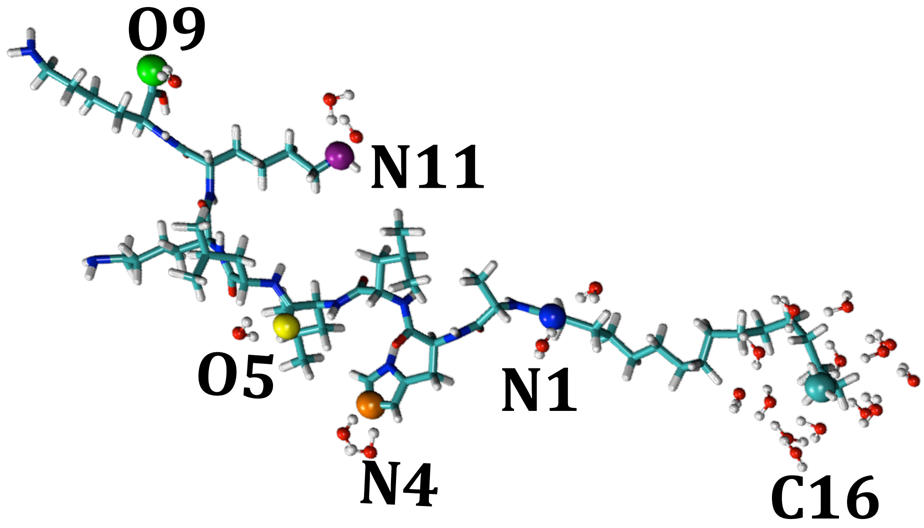

Supplementary Figure 1. Configuration of one of the PAs and water molecules, after ~150 ps, in the first hydration shell during Stage 1. Rearrangement of water molecules near hydrophilic groups and hydrophobic groups of the PAs to form hydrogen bonds with the PAs and with themselves can be seen, respectively. The C16 (in cyan), N1 (in blue), N4 (in orange), O5 (in yellow), O9 (in green), and N11 (in purple) atoms of hydrophobic tail, Alanine, Histidine, Leucine, and Lysine amino acids, respectively. Oxygen and hydrogen atom of water are shown in red and white, respectively.

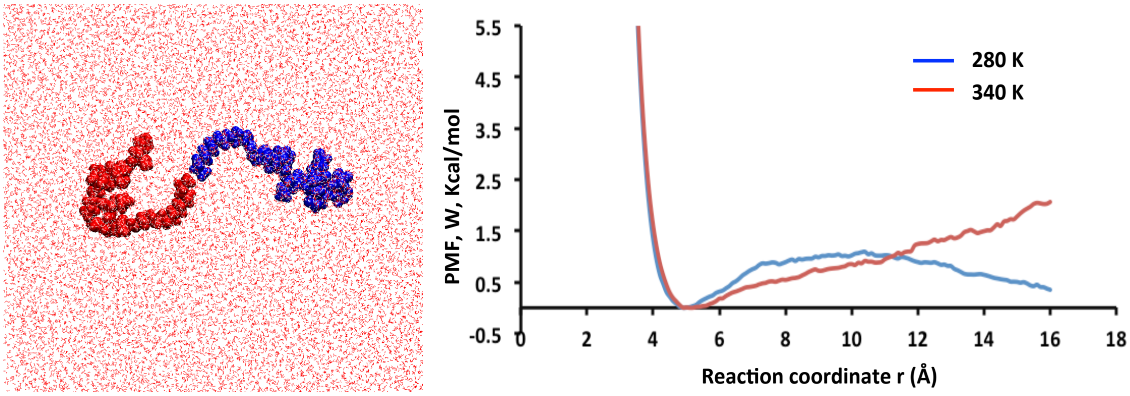

(a) (b)

Supplementary Figure 2. Potential of mean force (PMF) calculations on the solvated PAs. (a) Snapshot of initial configuration of PAs in water. PAs are shown in blue and red. (b) PMF as a function of interatomic distance between the hydrophobic groups of PAs at 280 K and 340 K.

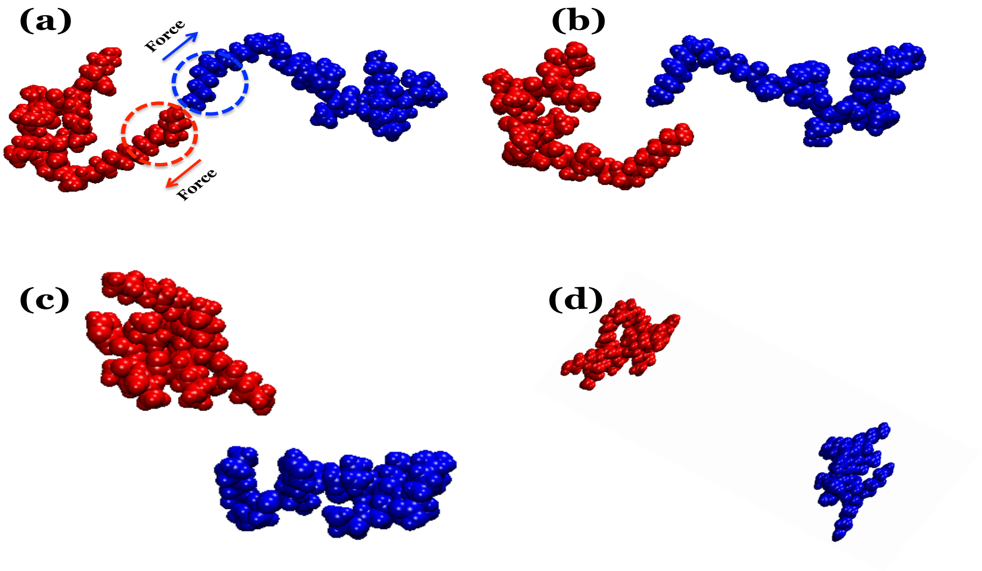

Supplementary Figure 3. Simulation snapshots during PMF calculations with increase in simulation time. Snapshots are shown at (a) t=0 ns and (d) t=10 ns. Blue and red circles and arrow show the reaction coordinates and direction of applied force on two peptides.

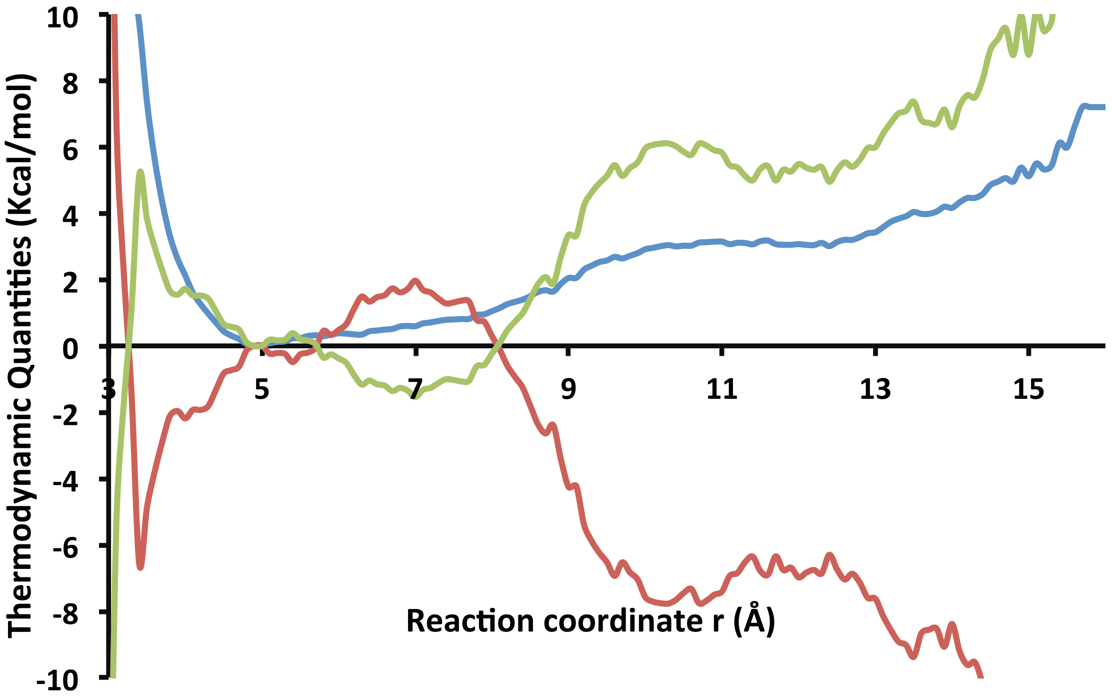

Supplementary Figure 4. Enthalpic and entropic contributions during the self-assembly of PAs. Enthalpic contribution, $\Delta H(r)$, (green line) and entropic contribution, $-T\Delta S(r)$, (red line) to the potential of mean force, $\Delta W(r)$, (blue line) are shown for a pair of PAs in water at 280 K and 1 atm pressure.

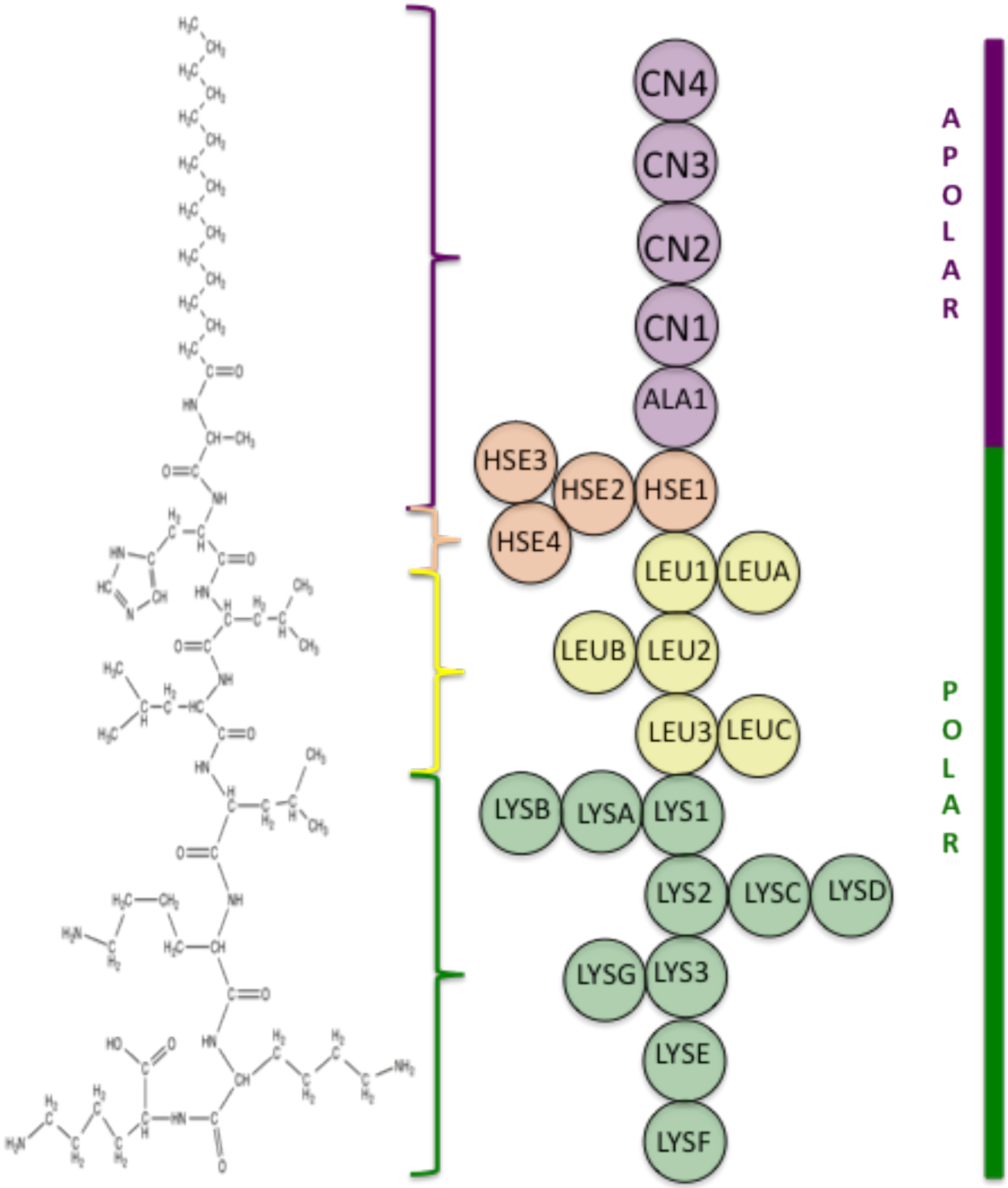

Supplementary Figure 5. Schematic structure of atomistic and CG model of PA molecule. Supplementary Table 3 lists the details of each bead of the CG model.

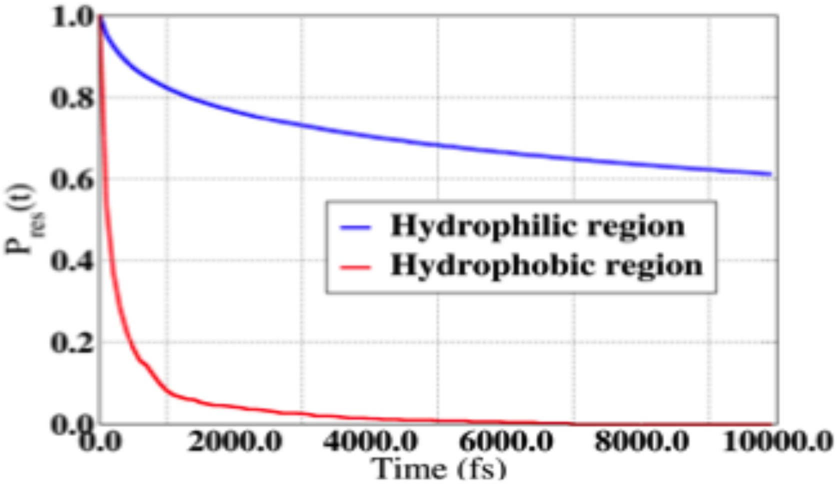

Supplementary Figure 6. Residence time probability ($P_{res}(t)$) of water molecules near hydrophilic and hydrophobic region of PA molecules. Note, alanine, histidine, leucine, and lysine amino acids were included in the definition of hydrophilic region of the PA and hydrophobic region is consisting of aliphatic chain. $P_{res}(t)$ was defined as the probability of continuous existence a water molecule in one of the above defined regions for the time interval t_0 and $t + t_0$. $P_{res}(t)$ was averaged over several different simulation frames that were collected in intervals of 1 ps over a time frame of last ~10 ns of ~150 ns simulation run of stage 3.

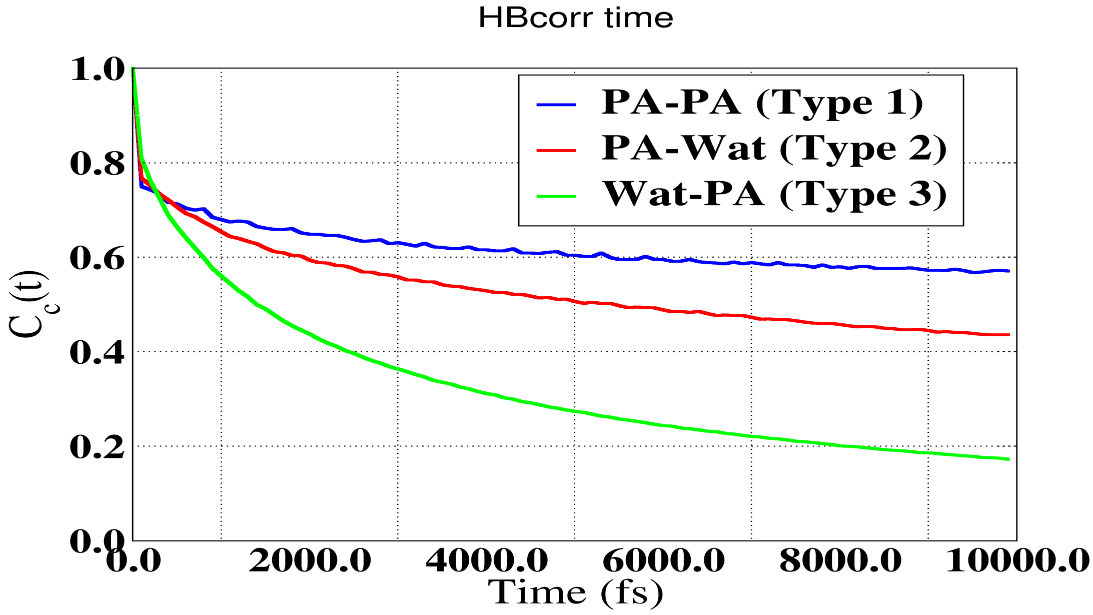

Supplementary Figure 7. Hydrogen bond correlations for PA and PA (type 1), PA and water (type 2), and water and PA (type 3) by continuous definition, at 300 K.

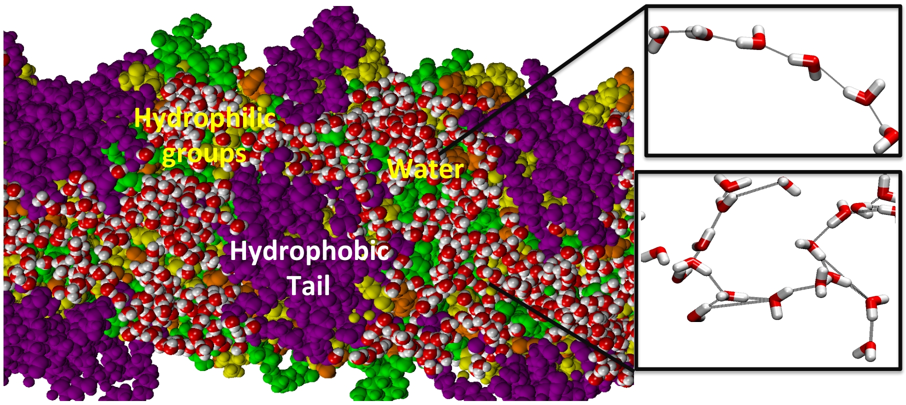

Supplementary Figure 8. Snapshot showing ordering of water in the fiber network at ~150 ns of simulation in stage 3. Black lines in enlarged snapshots show the hydrogen bonds between water molecules.

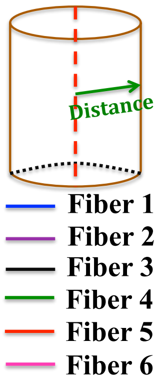

Supplementary Figure 9. Schematic representation of the radius of the cylindrical shape of self-assembled PA.

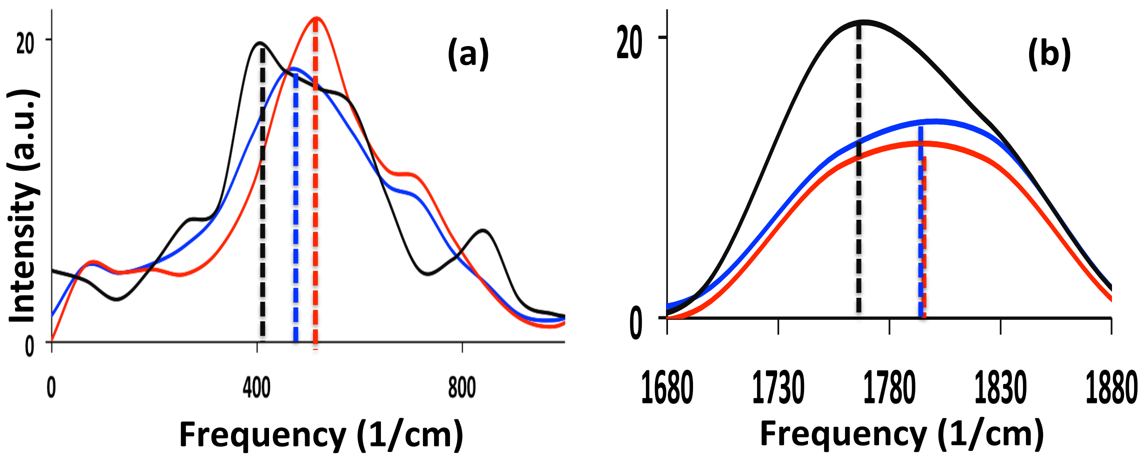

Supplementary Figure 10. The libration and bending band of the vibrational spectra for hydrogen of water molecules at the end of stage 1, at the end of stage 3, and bulk water. The black, blue, and red lines represent bulk water, proximal water at the end of stage 1, and proximal water at the end of stage 3, respectively.

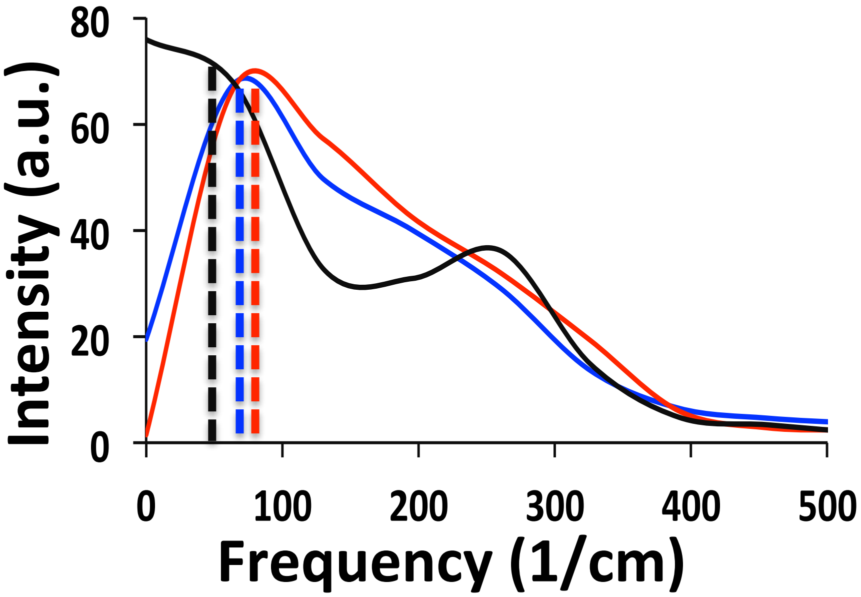

Supplementary Figure 11. The vibrational spectra for oxygen of water molecules at the end of stage 1, at the end of stage 3, and bulk water. The black, blue, and red lines represent bulk water, proximal water at the end of stage 1, and proximal water at the end of stage 3, respectively.

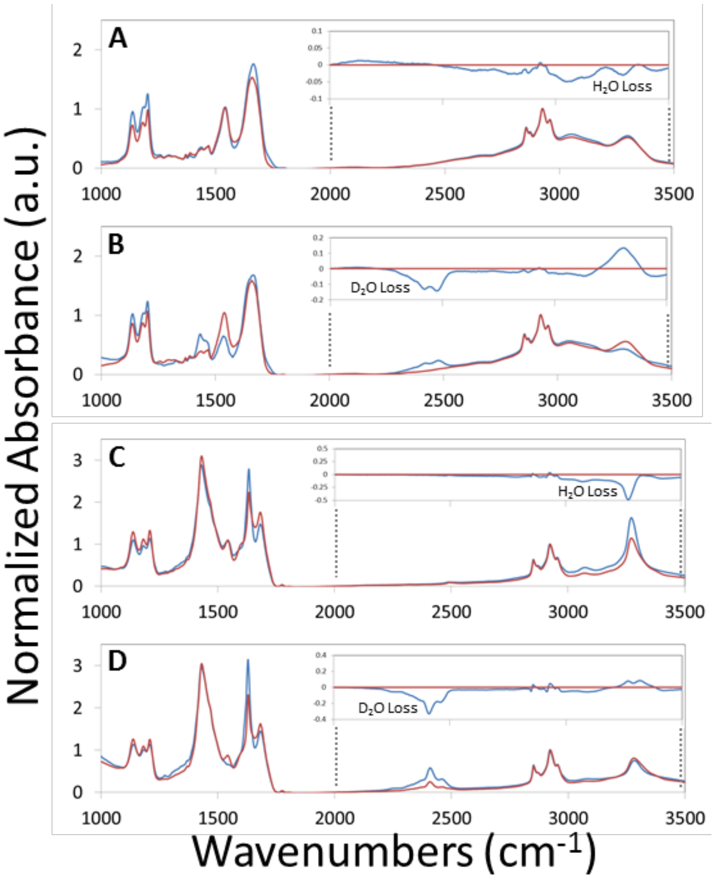

Supplementary Figure 12. Normalized FTIR Spectra of c16-AHL3K3-CO_2H. FTIR spectra are obtained for dried films on CaF_2 from (A) H_2O, (B) D_2O, (C) 10 mM NH_4OH, and (D) 10 mM ND_4OD. Freshly cast samples ('wet') are in blue while vacuum oven (100°C) dried samples ('dry') are in red. The inset in each panel is the difference spectrum of 'wet' – 'dry' from 2000 – 3500 cm^{-1}.

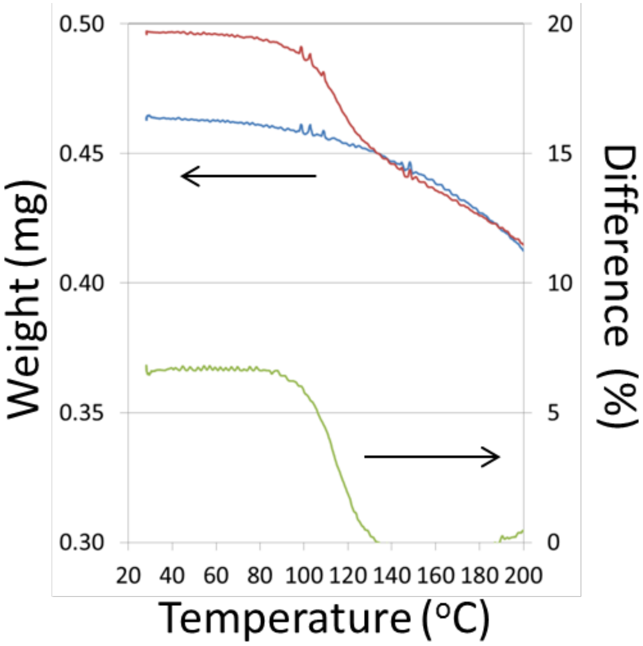

Supplementary Figure 13. Determination of water content in dried films. Thermogravimetric analysis from 25 – 200°C of c16-AHL$_3$K$_3$-CO$_2$H dried film from water (blue) and c16-AHL$_3$K$_3$-CO$_2$H dried film from fibers, 100 mM NH$_4$OH (red), left – axis. Percentage of water trapped in the fibrous assembly determined by plotting the percent difference in mass of the two films (green), right – axis.

Supplementary Table 1. Temporal evolution of the order parameters within 5 Å of the hydrophilic groups of PAs during initial 200 ps of 150 ns simulation run of stage 1.

Time (ps)	Translational order	Tetrahedral order
25	1.29±0.02	0.66±0.02
40	1.32±0.05	0.66±0.02
60	1.40±0.05	0.65±0.01
100	1.40±0.04	0.63±0.03
150	2.05±0.08	0.55±0.01
200	2.45±0.06	0.54±0.01

Supplementary Table 2. Temporal evolution of the order parameters within 5 Å of the hydrophobic groups of PAs during initial 200 ps of 150 ns simulation run of stage 1.

Time (ps)	Translational order	Tetrahedral order
25	2.78±0.05	0.52±0.01
40	2.72±0.06	0.52±0.01
60	2.74±0.09	0.51±0.01
100	2.62±0.03	0.54±0.02
150	2.60±0.03	0.59±0.01
200	1.30±0.12	0.66±0.02

Supplementary Table 3. Martini atom types and charges for the different coarse-grained bead used in this study. See Supplementary Figure 5 for mapping of various bead names.

Name	Amino Acid	Charge	CG Type
CN4		0	C1
CN3		0	C1
CN2		0	C1
CN1		0	NA
ALA1	Alanine	0	P4C
HSE1	Histidine	0	P5C
HSE2		0	SC4H
HSE3		0	SP1H
HSE4		0	SP1H
LEU1	Leucine	0	P5C
LEUA		0	AC1L
LEU2		0	P5C
LEUB		0	AC1L
LU3		0	P5C
LUC		0	AC1L
LYS1	Lysine	0	P5C
LYSA		0	C3K
LYSB		0	P1K
LYS2		0	P5C
LYSC		0	C3K
LYSD		0	P1K
LYS3		0	P5C
LYSE		0	C3K
LYSF		0	P1K
LYSG		0	P1

Supplementary Table 4. Spatial variation of translational and tetrahedral order parameters near the hydrophilic region during last 10 ns of 150 ns simulation run of stage 3.

Region	Translational order	Tetrahedral order
R< 3 Angstrom	2.69	0.51
10<R<3 Angstrom	1.82	0.59
R> 10 Angstrom	1.29	0.66

Supplementary Discussion

Water Ordering Around Peptide Amphiphile During Stage 1

The water molecules near the N1, N4, O5, O9, and N11 atoms of Alanine, Histidine, Leucine, and Lysine amino acids, respectively orient themselves to form hydrogen bond with these polar atoms as shown in Supplementary Figure 1. Strong solvation around the hydrophobic region of the PAs hydrophobic tail can also be seen during initial ~150 ps of simulations. This solvation of water molecules near the hydrophobic tail would lead to formation of water-cage-like structure around these hydrophobic groups due to lack of ability of these non-polar hydrophobic groups to form hydrogen bonds with water.

Order Parameters

Supplementary Table 1 and Supplementary Table 2 show the translational and tetrahedral order parameters near hydrophilic and hydrophobic groups of fibers, respectively, during the initial 200 ps of stage 1. We find that the translational and tetrahedral order of water increases and decreases, respectively, with increase in simulation time and approach the values of bulk water. Note, the bulk water has a translational order of ~1.2 and tetrahedral order parameter of ~0.68.[3] This suggests that water molecules near hydrophobic groups of PAs are ordered in very initial stages of simulations (< 150 ps). On the other hand water molecules near hydrophilic groups the translational and tetrahedral order of water increases and decreases, respectively. This suggests increase in ordering of water molecules near hydrophilic groups with increase in simulation time.

Potential of Mean Force of PA Aggregation

The entropic contribution, $-T\ \Delta S(r)$ to the PMF, $\Delta W(r)$, at 280 K is obtained through supplementary equation 5. The entropic ($-T\ \Delta S(r)$) and enthalpic ($\Delta H(r)$) contributions to the PMF at 280 K are shown in Supplementary Figure 4 along with the PMF. The entropic and enthalpic contributions of the free energy act in opposite direction to one another, and the relative proportion of the two contributions depend on the distance between the two PA molecules. Similar observations has been reported in prior studies both at nano and macro-scales.[5, 6]

As can be seen in Supplementary Figure 4 the enthalpic and entropic ($T\Delta S(r)$) contributions strongly depend on the reaction coordinates or the distance between the hydrophobic groups of PAs. At distance, r ≤ ~4.9 Å the entropic contribution increases (decrease in $-T\Delta S(r)$) and enthalpic contribution decreases. This suggests that the entropic contributions are much favorable as compared to the enthalpic contributions in stabilizing the contact pair state. With increase in the distance between two PA molecules (r>~6 Å and r<~8.5 Å), the stability of the first solvent separated state is mostly determined by the steady enthalpic effect. On the other hand the entropic contributions are relative smaller at this state (r>~6 Å and r<~8.5 Å). This could be due to the release of the highly structured water near PAs to the bulk. At higher distances (r>8.5 Å) we find that the entropic contribution of the system increases (decrease in $-T\Delta S(r)$ as shown in Supplementary Figure 4). Thus, the separated PA molecules are well stabilized by entropic contributions.

<u>Residence Time Probability of Water</u>

Supplementary Figure 6 shows the $P_{res}(t)$ for water molecules in hydrophilic and hydrophobic region of PA molecules. The water molecules near hydrophilic region reside for longer time as compared to water molecules near hydrophobic region. This shows that structure of water molecules near hydrophilic groups is more stable as compared to the water molecules near hydrophobic region. This can be attributed to the presence of water

molecules in the PA network that form stronger hydrogen bonds with the amino acids of PA, assisting water molecules to form stable network near hydrophilic region.

Hydrogen Bond Characteristics

In the systems studied, the hydrogen bonds were classified into three types based on donor-acceptor pair: (1) PA((Npa-)Hpa) – PA(Opa(=Cpa)), (2) PA((Npa-)Hpa) – water(Ow), and (3) water((Ow-)Hw) –PA (Opa(=Cpa)). The results of these analyses by the definition of continuous autocorrelation function, is shown in Supplementary Figure 7.

In the case of type (1) hydrogen bonds, both the donor and acceptor groups are on PA. The autocorrelation function decays slowly as compare to type (2) and type (3) hydrogen bonds. The slower decay observed in the case of type (1) hydrogen bonds can be attributed to the self-assembly of PAs which leads to strong hydrogen bonding among themselvels. Additionally, β-sheets formed between different PA molecules (Figure 2 in main manuscript) can also stabilize the PA structure and lead to increase in the hydrogen bond characteristics between PA and PA.Comparison of type (2) and (3) interactions, where the role of both donor and acceptor, respectively, is played by PAs suggests that the hydrogen correlation for type (2) decays slower as compared to hydrogen correlation for type (3). This slower decay dynamic of type (2) hydrogen bonds indicates that the hydrogen bond formed between hydrogen of amide groups of amino acids (Npa(-Hpa)) and oxygen atom of water (Ow) are more stable as compared the hydrogen bond formed between carbonyl oxygen (Opa) and hydrogen of water (Hw).

Order Parameter for Water in Stage 3

Supplementary Table 4 show the translational and tetrahedral order parameters near hydrophilic groups of hexagonally packed fibers during last 10 ns of stage 3. Supplementary Figure 8 show the snapshots of water molecules in the PA network at the end of stage 3. Both Supplementary Table 4 and Supplementary Figure 8 suggest that water in the hexagonally packed fibers is hydrogen bonded with each other to form

ordered network. Such extreme level of ordering of water is typical of that seen for water near solid interfaces.

Vibrational Spectra of Water

Supplementary Figure 10 shows the vibrational spectra of hydrogen of water molecules at the end of stage 1, at the end of stage 3, and bulk water. In the case of pure bulk water, similar to previous studies, the libration, bending, and stretching band shows characteristic peaks at ~390 cm^{-1}, ~1820 cm^{-1}, and ~3390 cm^{-1}, respectively.[8] The libration band for hydrogen of proximal water at the end of stage 3 (~520 cm^{-1}) shows a blue shift as compared to the proximal water at the end stage 1 (~460 cm^{-1}) and bulk water (~390 cm^{-1}) (Supplementary Figure 10 (a)). This suggests that at the end of ~150 ns of stage 3 due to increased ordering of water molecules the hydrogen bond network within water molecules is stronger as compared to the bulk water and proximal water at the end of stage 1 (Supplementary Table 4 and Supplementary Figure 8).[8] The bending band of hydrogen atom shows a blue shift for proximal water present at the end of stage 1 and stage 3 (1890 cm^{-1}) as compared to the bulk water (1760cm^{-1}) (Supplementary Figure 10 B). The blue shift in the O—H bending band in water molecules can be attributed to the increase in the strength of the hydrogen bonds in proximal water molecules with PA molecules or with itself.

Supplementary Figure 11 shows the vibrational spectrum for the oxygen atom of water molecules in bulk water and proximal water at the end of stage 1 and stage 3. The low-frequency (< ~600 cm^{-1}) end of the vibrational spectrum for oxygen atom of water shows two broad bands representing the O···O···O intermolecular bending motions of H-bonded molecules and the O···O intermolecular stretching mode at ~60 cm^{-1} and ~ 250–

300 cm^{-1}, respectively.[8] The peak for the bulk water observed at ~60 cm^{-1} shows a blue shift for proximal water present at the end of stage 1 and stage 3 at ~70 cm^{-1} and ~90 cm^{-1}, respectively. This blue shift suggests the reduction in the tetrahedral order and changes in the distribution of hydrogen bonds in ordered proximal water near PA molecules (Supplementary Table 4). In addition a shoulder for the O···O intermolecular stretching mode was observed at ~260 cm^{-1} for bulk water. This peak shows blue shift for the proximal water molecules present at the end of stage 1 and stage 3 at ~270 and ~ 320 cm^{-1}. This suggests that a stronger hydrogen-bonding network is present in proximal water near the PA molecules chain as compared to bulk water.[10]

Experimental Infra Red Spectra of Dried Sample

In an effort to remove confined water (both H_2O and D_2O samples) from the dried samples, the peptide material films were placed in a vacuum oven for 16 hrs, Supplementary Figure 12. The infrared spectra were recorded before and after vacuum drying. In the case of unassembled peptide at neutral pH, we observe a slight loss in absorption at ~3300 cm^{-1} in the case of H_2O but in the case of D_2O we observe a dramatic loss in 2420 and 2475 cm^{-1} with a rise at 3300 cm^{-1}. In the assembled fibers at high pH, we observe a loss of signal at 3270 cm^{-1} (2410 cm^{-1} for D_2O). We must acknowledge the difficulty in assigning the peaks when there are conflicting assignments of the amide A vibrations that overlap significantly with water vibrations as well as the near identical shifts upon deuteration of water or peptide. However, our results show a dramatic change in absorption in regions of the spectrum that are significantly influenced by vacuum treatment at elevated temperatures that suggest the removal of confined water and also follow the vibrational trends that we observed in our molecular dynamics simulations.

Thermogravimetric Analysis

TGA experiments suggest that ~0.3 mg water is trapped in the fibrous assembly (Supplementary Figure 13). We also dehydrated the sample with excessive vacuum application and found a decrease in the overall signal.

Sources of uncertainty in our model

The following are some of the important factors that one needs to consider while implementing a multi-stage couple all-atom coarse-grained MD approach:

Compatibility of all-atom and coarse-grained force-fields: It is very important that both all-atom and coarse-grained force-fields are compatible with each other and will preserve the structural and dynamical properties of the a given system throughout the course of they study. In the past, the combination of both CHARMM and MARTINI has been used successfully to study proteins and lipids.[12] Hence, in the present study we have utilized CHARMM and MARTINI force-fields for all-atom and coarse-grained models, respectively. Indeed, we find that at the end of final stage (described as stage 3 in original manuscript) after CG model is back mapped to all-atom model the nanofibers and their hexagonal packing remains intact this shows the compatibility of MARTINI and CHARMM force-fields. In addition, we have validated our simulation results with experimental results by comparing structural properties of both PA and solvent molecules.

Mapping Algorithm: Choice and accuracy of the mapping algorithm to transform an all-atom to a CG model and back mapping algorithm to go back to an all-atom model from a CG model also plays an important role in generating realistic starting configurations. In particular, unrealistic initial configurations or small errors in the input structure may cause MD simulations to become unstable or give unrealistic trajectories. Back mapping is a challenge since any given conformation of CG particles can be taken to represent an ensemble of conformations of the corresponding all-atom system (any set of states where the centers of mass of the component atoms for each bead correspond to the CG bead positions), and because switching to the all-atom system can likely cause unphysical change in the energy of the system due to the introduction of new interactions. Hence, while back mapping the MARTINI CG model to all-atom model, we transform all-atom structures by mapping the center of mass of the group of atoms represented by a single CG bead to that bead's location. To

capture the correct orientation of the atoms, we perform a local minimization using the BFGS algorithm. This can be conceptually interpreted as sampling the conformational space of the all-atom structure in the region consistent with the CG structure being converted. While this method is far too time-consuming, it is sufficient for recovering an energetically favorable all-atom snapshot from a CG simulation given that mapping the CG conformation to an all-atom conformation is nontrivial. Hence, one needs to be very careful while using coarse-graining methods and back mapping algorithms.

Solvent Density: The density of solvent molecules in different regions of the structure in coarse-grained and all-atom models need to be maintained. In the present study, the close packing of nanofibers can lead to trapped network of water molecules, which can significantly affect structural and dynamical properties of water molecules. For example, the residence time of water molecules, the diffusion, the tetrahedral ordering of water under confinement between two closely packed fibers can be significantly different that the bulk water. Hence, while reinserting water molecules in the simulation cell that is consisting of all-atom self-assembled structure of PA nanofibers, we maintain the higher density of ~1.1 - ~1.2 g/cc water between the two fibers. However, the overall water density of kept at 1 g/cc.

Supplementary Methods

<u>Calculations of Order Parameters</u>

We find that the water near PAs form ordered structure. We calculate the translation and orientational order parameters for water molecules. Here are the calculation details:

Translation order

The translational order parameter (t) is given by:[1]

$$t \equiv \int_0^{s_c} \left| g_{OO}(s) - 1 \right| ds$$

.... **Supplementary Equation 1**

where, $g_{OO}(r)$ = the oxygen-oxygen radial distribution function.

s (dimensional variable) $=r\rho^{1/3}$, r = radial distance that is scaled by the mean intermolecular distance $\rho^{-1/3}$ ($\rho=N/V$).

s_c= distances which are large enough to have $g(sc)\approx 1$. In this work, s_c~10 Å was chosen.

In an ideal gas, the RDF is equal to 1 and t=0. In a crystal, there is long-range order and $g_{OO}(r)=1$ over long distances, and order parameter t is large. The physical meaning of t is such that both positive and negative oscillations of $g_{OO}(r)=1$ and contribute equally to this quantity. In a system with long-range order e.g., a crystal, these oscillations persist over long distances, and hence t is large. The range of the translational order can vary from ~1.2 (for bulk water) to ~3.4 for hexagonal ice.

Orientational order (q)

We calculate the orientational order parameter q to evaluate the differences in the tetrahedrality of the structured water near PAs. The orientational parameter $q$describes the ability of the water to produce tetrahedral arrangements and can be given as:[2]

$$q \equiv 1 - \frac{3}{8}\sum_{j=1}^{3}\sum_{k=j+1}^{4}\left[\cos\theta_{jik} + \frac{1}{3}\right]^2$$ **.......................Supplementary Equation 2**

Where, θ_{jik}= angle formed by the lines joining the oxygen atom of a given molecule with that of each of the four nearest neighboring molecules. In this study, the ensemble average is obtained over all the molecules and the timeframes of the simulation. The value of q would effectively range from 0, in a random distribution of molecules to 1, in a perfect tetrahedral arrangement. Note that that the value of this order parameter is not

subjected to the choice of any particular local coordinate system in the reference molecule.

Calculations of Potential of Mean Force of PA Aggregation

The PMF between two PAs can be written as a function of the separation between them, W = f(r). The mean force, F(r) between these two atoms is then defined as:

$$\boldsymbol{F(r) = -\frac{d}{dr}[W(r)]}$$ ……… **Supplementary Equation 3**

The PMF between two PAs can be calculated by using following equation:

$$\boldsymbol{W = f(r) = -k\,T\,lnP(r) + W_0}$$ ……… **Supplementary Equation 4**

Where, r = distance between the hydrophobic tails of two different PAs.

k = Boltzmann constant;

T = Temperature in Kelvin;

P(r) = Probability of finding the system with specified r;

W_0 = Constant.

W = Effective interaction between two PAs averaged over the conformations of all other components in the system.

We employed constrained MD simulations and adaptive biasing method reference to calculate the potential of mean force (PMF).[4] Molecular dynamics simulations were performed using NAMD version 2.9. In the initial stage of equilibration both the PA molecules were kept in a place by fixing the position of all PA atoms in presence of mobile water. The system was equilibrated for 5 ns under NPT ensembles at 280 K and 1 atm. For all the calculations, two different temperatures (280 K and 340 K) were maintained using Langevin dynamics and constant pressure was set at 1.0 atm using the Nose–Hoover algorithm. The barostat oscillation time scale for the Langevin piston was kept at 50 fs. A time step of 1.0 fs was used for the integration of Newton's equation of

motion. Periodic boundary conditions were used in all the three spatial coordinates. Long range electrostatic interactions were calculated using the Particle-Mesh Ewald algorithm. A switching function was applied for all Lennard-Jones interactions at 12.5 Å for a 14.0 Å cut-off. With this option turned on, a smooth switching function will be used to truncate the van der Waals potential energy smoothly at the cutoff distance. A switch distance of 10 Å was used for activating the splitting function for electrostatic and van der Waals calculations. A pair list distance of 14 Å was used to include the pairs of atoms for which electrostatics and van der Waals interactions were calculated.

The PMF was calculated with the adaptive biasing force (ABF) method from a 10 ns MD simulation trajectory in the isothermal-isochoric (NVT) ensemble. The reaction coordinate for the determination of free energy changes was defined as the distance between groups of atoms on the hydrophobic tails of PA molecules. Over the course of simulation, the reaction coordinate spanned a distance of 16.0 Å between the selected atoms on the hydrophobic tails. A single 10 ns ABF run was performed spanning the complete reaction pathway from 0.0 Å to 16.0 Å after equilibration of the system. Force statistics were stored in bins of width 0.05 Å. The biasing force was applied after 500 samples were collected in each bin. Statistical errors were estimated from the standard deviation of the predicted free energies generated from these simulations.

Supplementary Figure 2 (a) and (b) shows the initial configuration and PMF profile for the PA system.

To calculate the entropy we used the finite difference temperature derivative of the PMF, $W(r)$ at each inter-atomic distance between the group of atoms on hydrophobic tails, r, as:

$$-T\Delta S(r) = T[\frac{W(r,(T+\Delta T)-W(r,(T-\Delta T)}{\Delta T}]$$ .... **Supplementary Equation 5**

Where, ΔT is the temperature difference.

The enthalpy contribution to the free energy, $H(r)$, can then be obtained from entropy $S(r)$ and the PMF, $W(r)$ at temperature T as:

$$\Delta \boldsymbol{H}(\boldsymbol{r}) = \Delta \boldsymbol{W}(\boldsymbol{r}, \boldsymbol{T}) + \boldsymbol{T} \Delta \boldsymbol{S}(\boldsymbol{r})$$ **……… Supplementary Equation 6**

Here, values of T and ΔT were chosen to be 280 and 40, respectively.

<u>Coarse-grained Model of PAs</u>

The MARTINI CG model has been previously used to study the self-assembly of peptides.[7] This model uses a four-to-one mapping scheme, wherein four heavy atoms (e.g. C, O, N) and their associated hydrogen atoms are represented as a single CG bead. Four water molecules are also mapped to form one water bead and are treated explicitly. The intra-molecular bonded interactions are defined by the bond, angle, and dihedral energy functions. Both intra- and inter-molecular non-bonded interactions are represented by the Lennard-Jones and Coulomb functional forms. Supplementary Figure 5 and Supplementary Table 3 lists the details of the mapping of the CG beads of PAs to the MARTINI model. CG MD simulations were performed with the NAMD simulation package with a timestep of 30 fs in the NPT ensemble and the total simulation time was 16 μs.[8] The pressure and temperature are controlled by employing the Langevin barostat and thermostat at 1 bar and 340 K, respectively. The neighbor list cutoff of r_{cut} = 2.0 nm was used and the list was updated every 10 steps. Simulation trajectories including coordinates and velocities of atoms were saved every 5 ns for the trajectory analysis.

In the third stage of self-assembly process we removed all the CG water molecules from the simulation cell. This was followed by back-mapping of the CG model of PA to fully-atomistic model by using VMD's Coarse Grain Builder tool.[9] This all atom structure was further solvated by randomly distributed modified TIP3P water molecules.

Simulations were carried out using NAMD simulation package using NPT ensemble for 150 ns with 1 fs timestep. The parameters for the NPT ensemble are identical to the one described in first stage of the self-assembly simulations.

Calculation of Residence Time Probability P_{res} (t)

Residence probability ($P_{res}(t)$) of water molecules near the hydrophobic tail and amino acids was calculated at the end of stage 3. Water molecules were divided into two different regions: (1) hydrophilic water molecules (water molecules located within 5.5 Å of hydrophilic groups) and (2) hydrophobic water molecules (water molecules located within 5.5 Å of hydrophobic groups). Note, alanine, histidine, leucine, and lysine amino acids were included in the definition of hydrophilic region of the PA and hydrophobic region is consisting of aliphatic chain. $P_{res}(t)$ was defined as the probability of continuous existence a water molecule in one of the above defined regions for the time interval t_0 and $t + t_0$.[9] $P_{res}(t)$ was averaged over several different simulation frames that were collected in intervals of 1 ps over a time frame of last ~10 ns of ~150 ns simulation run.

Hydrogen Bonding Criteria

Hydrogen bonding characteristics were evaluated using following geometric criteria for hydrogen bonding:[10]

$$R_{OO} \leq 3.6 \text{ Å}$$
$$R_{OH} \leq 2.45 \text{ Å}$$
$$Ø \leq 30°$$

......................................Supplementary Equation 7

In supplementary equation 7, R_{OO} and R_{OH} are the distances between oxygen and oxygen and between oxygen and hydrogen of water 1 and 2, respectively, which are involved in hydrogen bond formation. The angle between oxygen of water 1 and oxygen & hydrogen of water 2 ($O_1\cdots O_2–H_2$) can be defined by angle ϕ. In the present study, the

cut-off distances, R_{OO} and R_{OH} were defined based on the first minimum in the corresponding radial distribution functions of pure water.

Here, total three types of hydrogen bonds are possible. One hydrogen bond between PA and PA *(Type 1)* and two types of hydrogen bonds are possible between peptide and water **(1)** bond between oxygen (Opa) or nitrogen (Npa) atoms of alanine, histidine, leucine, and lysine amino acids and water hydrogen (Hw) *(Type 2)* and **(2)** bond between hydrogen of amino acid groups (Hpa) and water oxygen (Ow) *(Type 3)*. Similar to our previous study conducted for polymer-water systems, we have defined the geometric criteria for hydrogen bonds to exist between PA and PA, between PA and water, and between water and PA based on the location of the 1st peak of the RDF for respective donor and acceptor atoms.[10] For example, to define the hydrogen bonds between PA and water, where PA is donor [mainly hydrogen atoms bonded to nitrogen atoms (Npa-Hpa)] and water [oxygen of water (Ow)] is acceptor, the first distance criterion ($R_{Npa\text{-}Ow}$) requires the distance between Npa atom of PA and oxygen atom of water (ow) to be <= 4.2 Å (based on the RDF of Ow and Npa). The second distance criterion ($R_{Ow\text{-}Hpa}$) requires the distance between acceptor (Ow) and hydrogen (Hp) atom to be <= 2.45 Å (based on the RDF of Ow-Hpa) and the angle between acceptor-donor-hydrogen (Ow-Npa-Hpa) atom is required to be <30 °.

A hydrogen bond occupation number (S_{ij}) can be defined to distinguish between the hydrogen bonded and non-hydrogen bonded atoms.

$$\text{Hydrogen bond occupation number } (S_{ij}) = \begin{cases} 1 \text{ (hydrogen bonded)} \\ 0 \text{ (non-hydrogen bonded)} \end{cases}$$ **…. Supplementary Equation 8**

The S_{ij} that describes the existence or nonexistence of bonds between a selected donor-acceptor pair *ij (*supplementary equation 8*)*. A time-dependent autocorrelation function of this state variable S_{ij} can be defined to determine the stability of the hydrogen bonds. We use the continuous correlation functions to define the S_{ij}. In the case of continuous correlation, the hydrogen bond occupation number (S_{ij}) was allowed only one transition from 1 to 0 when the bond between atom *i* and *j* breaks for the first time. After the first breakage of hydrogen bonds between atom *i* and *j*, S_{ij} was never allowed to return to 1.

$$C_x(t) = \left\langle \frac{\sum_{ij} S_{ij}(t + t_0) \,.\, S_{ij}(t_0)}{\sum_{ij} S_{ij}(t_0) \,.\, S_{ij}(t_0)} \right\rangle$$

…………………..Supplementary Equation 9

where, x = continuous (*c*) autocorrelation function.

Calculations of Vibrational Spectra of Water

The vibrational spectra for water molecules were calculated by Fourier transforming of the velocity autocorrelation function (VAF) of water molecules.[11] This provide the density of states (DOS) spectral frequencies characteristic of various intra-molecular vibrational modes. The vibrational spectra can be calculated as:[11]

$$G(\omega) = \frac{1}{K_B T} \sum_j m_j \left[\frac{1}{2\pi} \int_{-\infty}^{\infty} dt \ \exp(-i\omega t) < v_j(0).v_j(t) > \right]$$ **…………Supplementary Equation 10**

where, $v_j(t)$ represents the velocity at time *t* for atom type *j* and the angular brackets denote an average over the ensemble and over time 0. This power spectrum will also contain anharmonic contributions. A separate MD simulation was conducted on the final configuration generated at the end of the production run of 150 ns (both at the end of stage 1 and at the end of stage 3) to calculate the vibrational spectra. Simulations were carried out with a time-step of 0.5 fs at 300 K for 100 ps with velocities for each atoms

being stored at every time-step. Water molecules within 5 Å of PA atoms were selected as proximal water molecules. Similarly, we have also calculated the vibrational spectra for bulk water at 300 K to facilitate comparison with the spectra obtained for proximal water.

Supplementary References:

1. J. R. Errington and P. G. Debenedetti, Relationship between structural order and the anomalies of liquid water, *Nature*, 409, 318-321 (2001)
2. N. Giovambattista, P. G. Debenedetti, F. Sciortino and H. E. Stanley, Structural order in glassy water, *Phys. Rev. E: Stat., Nonlinear, Soft Matter Phys.*, 71, 061505 (2005)
3. Z. Yan, S. V. Buldyrev, P. Kumar, N. Giovambattista, P. G. Debenedetti and H. E. Stanley, Structure of the first-and second-neighbor shells of simulated water: Quantitative relation to translational and orientational order*, Phys. Rev. E: Stat., Nonlinear, Soft Matter Phys.*, 76, 051201 (2007)
4. J. Henin, G. Fiorin, C. Chipot, M. L. Klein, Exploring multidimensional free energy landscapes using time-dependent biases on collective variables, *J Chem Theory Comput* 2010, *6*, 35-47.
5. N. Choudhury and B. M. Pettitt, Enthalpy-Entropy contributions to the potential of mean force of nanoscopic hydrophobic solutes, *J. Phys. Chem. B*, 110, 8459-8463 (2006)
6. N. M. Uddin, F. M. Capaldi, B. Farouk, Molecular dynamics simulations of the interactions and dispersion of carbon nanotubes in polyethylene oxide/water systems, Polymer, 52, 288-296 (2011)
7. L. Monticelli Luca, S. K. Kandasamy, X. Periole, R. G. Larson, P. D. Tieleman, S-J Marrink, The MARTINI coarse-grained force field: extension to proteins, *J. Chem. Theor. Comp.*, 4, 819-834 (2008)
8. J. C. Phillips, R. Braun, W. Wang, J. Gumbart, E. Tajkhorshid, E. Villa,C. Chipot, R. D. Skeel, L. Kalé, and K. Schulten, Scalable molecular dynamics with NAMD, *J. Comput. Chem.*, 26, 1781-1802 (2005)
9. A. Y. Shih, A. Arkhipov, P. L. Freddolino, and K. Schulten, Coarse grained protein−lipid model with application to lipoprotein particles, *J. Phys. Chem. B*, 110, 3674-3684 (2006)
10. S. A. Deshmukh, S. K. R. S. Sankaranarayanan, K. Suthar, and D. C. Mancini, Role of solvation dynamics and local ordering of water in inducing conformational transitions in Poly(*N*-isopropylacrylamide) oligomers through the LCST, *J. Phys. Chem. B*, 116, 2651-2663 (2012)
11. S. A. Deshmukh, S. K. R. S. Sankaranarayanan, and D. C. Mancini, Vibrational spectra of proximal water in a thermo-sensitive polymer undergoing conformational transition across the lower critical solution temperature*, J. Phys. Chem. B*, 116, 5501-5515 (2012)
12. T. A. Wassenaar, K. Pluhackova, R. A. Böckmann, S. J. Marrink, and D. P. Tieleman, Going backward: A flexible geometric approach to reverse transformation from coarse grained to atomistic models, *J. Chem. Theory Comput.* 10, 676-690 (2014)
